# Emerging nanotechnological strategies for delivering bioactive components from botanical drugs and traditional Chinese medicine in atherosclerosis therapy: from formulation to mechanism

**DOI:** 10.3389/fphar.2026.1792534

**Published:** 2026-05-26

**Authors:** Guo Zhili, Guan Zhimin

**Affiliations:** Jiaxing Hospital of Traditional Chinese Medicine, Jiaxing University, Jiaxing, China

**Keywords:** atherosclerosis, botanical drug, targeted therapy, TCM metabolites, TCM nano-preparations

## Abstract

Atherosclerosis (AS), the primary pathological basis of global cardiovascular disease mortality, is undergoing a paradigm shift in treatment strategy, from risk factor management toward precise plaque intervention. Nano-formulations are a major research focus in pharmaceutics. The “nanoization” of traditional Chinese medicine (TCM) has opened new avenues for innovation, not only altering the scale of TCM applications but also expanding their therapeutic scope. TCM nano-formulations can preserve the “multi-target, multi-level” characteristics inherent to TCM therapy while addressing low oral bioavailability and inadequate tissue targeting of active metabolites. These nanoparticles are typically produced by combining extracts or active compounds from botanical drugs or medicinal plants with nanocarriers. Compared with conventional botanical drug preparations, they can markedly improve bioavailability and stability, reduce toxic side effects, and preserve the intrinsic properties of the medicinal components. This article systematically reviews common types of TCM nano-formulations, including nanoemulsions, nanosuspensions, and nanoliposomes, and discusses recent advances in their application to AS. Furthermore, it analyzes bioinspired nano-delivery systems, such as platforms integrating anti-AS peptides, HDL, platelet membranes, or macrophage membranes with natural metabolites possessing anti-inflammatory or antioxidant activities. These systems exert anti-AS effects through mechanisms including reducing cholesterol levels, promoting cholesterol efflux, and driving macrophage repolarization toward an anti-inflammatory phenotype. Their successful application depends on precise control of nanoparticle size and rational delivery-system design. Nanoization not only enhances drug solubility and stability but also improves therapeutic efficacy and reduces toxicity while maintaining the natural pharmacodynamic profile. Future research may focus on developing oral nano–drug delivery systems, the expansion of targeting strategies by leveraging the homing properties of other immune cells to inflammatory sites, the creation of theranostic platforms using natural products or biologics, and employing microfluidic technologies to enable synergistic co-delivery and enhanced efficacy of dual therapeutic agents. These efforts are expected to provide important insights for research and development (R&D) and clinical translation of new therapies for cardiovascular diseases.

## 1 Introduction

Atherosclerosis (AS), the primary pathological basis of global cardiovascular disease mortality (accounting for 31% of all-cause deaths) (Song et al., 2025; Zheng et al., 2025), has undergone an evolution in mechanistic understanding from the traditional “lipid infiltration theory” to a “multi-omics regulatory network” perspective (Moore et al., 2018). This emerging paradigm encompasses areas such as epigenetic modifications and single-cell–resolved microenvironmental interactions (Sun et al., 2025). Although PCSK9 inhibitors can reduce LDL-C levels (Ge et al., 2025), residual risk persists, and a proportion of patients still develop cardiovascular complications (Wang et al., 2020). Nano-formulations, leveraging advanced nano-delivery technologies, demonstrate considerable therapeutic potential. For example, a biomimetic pHDL nanocarrier loaded with ***salvianolic acid B*** enhances cholesterol efflux from plaques by mimicking reverse cholesterol transport (Shan B. et al., 2025). Many plant-derived drugs are already used clinically, including ***artemisinin*** (a metabolite from sweet wormwood for malaria), ***berberine*** (for diarrhea), and paclitaxel (as a chemotherapeutic agent) (Tu, 2011; Song et al., 2020; Xu et al., 2025). Furthermore, natural metabolites such as berberine (Ma et al., 2021; Ochin and Garelnabi, 2018), ***artesunate*** (Cen et al., 2023), ***American ginseng saponins*** (Ma et al., 2022), ***curcumin*** (Lv et al., 2020; Ouyang et al., 2022; Meng et al., 2019), and ***epigallocatechin-3-gallate (EGCG)*** (Saadh et al., 2025; Yamagata, 2020) have demonstrated well-defined anti-AS mechanisms. For example, ***curcumin*** promotes cholesterol efflux in THP-1 macrophages and upregulates ABCA1 expression via the miR-125a-5p/SIRT6 axis, thereby preventing AS (Tan et al., 2021). ***EGCG*** protects endothelial cells from homocysteine-induced apoptosis by activating the SIRT1/AMPK and Akt/eNOS signaling pathways, thereby mitigating AS pathogenesis (Pai et al., 2021). These natural agents have been investigated for treating hypercholesterolemia and AS. The chemical diversity of medicinal-plant metabolites and their broad applicability enable innovative products with the potential for fewer side effects than existing drugs. Whereas the compounds discussed in this article (e.g., curcumin, danzhenyuan IIA) are classified as natural products in plant chemistry, they are often used within TCM theory to treat specific syndromes and therefore have an important TCM context. Accordingly, this review focuses on active components derived from TCM and examines how to retain their “TCM attributes” within nano-delivery systems. However, the clinical translation of TCM-derived therapeutics is often limited by restricted administration routes, poor aqueous solubility, low bioavailability, and insufficient targeting (Chen et al., 2023a). Nanotechnology is being applied to address these limitations through high permeability, improved targeting, and controlled release. Two major nanotechnology applications in botanical drug research are nano-sizing and nano-delivery systems. Drug nanoization can enhance solubility and stability, preserve key properties of phytomedicines, and simultaneously increase efficacy while reducing toxicity (Wang et al., 2019). Furthermore, TCM nano-delivery systems enable more precise drug delivery and controlled release, thereby enhancing therapeutic efficacy and minimizing side effects through synergistic interactions with the carriers (Zhang et al., 2023). To date, substantial progress has been made in herbal nano-research. This review summarizes recent research hotspots and advances in applying botanical drug nano-formulations to AS treatment and systematically describes how nanotechnology can address the solubility–targeting–efficacy limitations of TCM components.

## 2 Methods

Search Strategy: A systematic literature search was conducted in March 2025 across four biomedical and pharmaceutical databases: PubMed, Web of Science Core Collection, Scopus, and ScienceDirect. Supplementary searches were performed using Google Scholar to identify specific documents, and the reference lists of included articles were manually screened to capture additional relevant studies. Search terms were developed using keywords, truncations, and Boolean combinations: [(“traditional Chinese medicine” OR “TCM” OR “herbal medicine” OR “botanical drug”) AND (“nano” OR “nanoparticle” OR “nanocarrier” OR “drug delivery system”) AND (“atherosclerosis” OR “cardiovascular” OR “plaque”)]. Only articles published in English were considered, with no restrictions on publication date.

### 2.1 Inclusion and exclusion criteria

Inclusion criteria were: (1) studies focusing on nano-formulations of active ingredients derived from TCM (including single compounds, active fractions, or formulas); (2) studies involving the design, characterization, and *in vitro*/*in vivo* evaluation of nano-delivery systems; (3) studies targeting AS or related pathological processes (e.g., endothelial injury, lipid deposition, inflammatory responses, plaque formation). Exclusion criteria were: (1) studies addressing only the pharmacological activities of TCM components without nano-delivery systems; (2) studies focusing solely on the synthesis and characterization of nanomaterials without application to TCM-component delivery; (3) non-original research such as reviews, conference abstracts, commentaries, and editorials; (4) non-English publications.

Study Selection and Data Extraction: All retrieved records were imported into EndNote for duplicate removal. Two independent reviewers screened titles and abstracts using the predefined inclusion and exclusion criteria. Articles deemed potentially eligible were then evaluated by full-text review. Discrepancies during study selection were resolved through discussion or, when necessary, consultation with a third reviewer. From the final set of included studies, the following data were extracted: first author, publication year, TCM component name, nanocarrier type, preparation method, characterization parameters, *in vitro*/*in vivo* models, key findings, and proposed mechanisms.

Quality Appraisal: Due to the absence of universally accepted quality assessment tools or reporting guidelines for systematic reviews in the interdisciplinary field of TCM nanomedicine, no formal quality appraisal was performed. This limitation is common in reviews at the interface of nanomedicine and traditional medicine. Instead, we qualitatively evaluated the contribution of each study based on the completeness of the work, methodological rigor, and depth of discussion of core issues.

Data Analysis and Synthesis: A qualitative content analysis approach was adopted to inductively categorize and synthesize findings from the included studies. The analysis was organized around the core theme of “nano-delivery systems for TCM components in AS therapy: from formulation to mechanism.” Sub-themes included: (1) classification and characteristics of active TCM components; (2) types of nanocarriers and associated delivery strategies; (3) targeting requirements and principles for matching carriers across pathological stages; (4) challenges and design strategies for multi-component co-delivery; (5) *in vivo* fate and therapeutic mechanisms of nano-formulations; (6) challenges in clinical translation and future directions.

## 3 Synthetic nanocarriers

**FIGURE 1:**
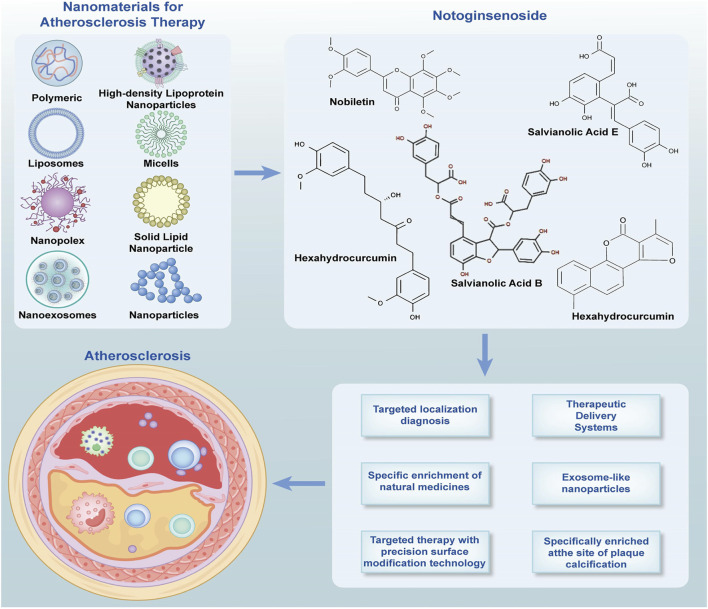
Main types and application value of TCM nano-formulations.

In recent years, advances in materials science, pharmaceutics, and pharmacokinetics have diversified drug dosage forms. Novel formulations have reshaped how drug action is conceptualized and have expanded the conventional boundaries of drug application. “Nanodrugs,” produced through “nanoization,” involve processing drugs and carrier materials into drug crystals or drug-loaded particles with sizes of 10–1,000 nm, thereby enabling enhanced targeting and distinct pharmacological profiles compared with conventional formulations (Yao et al., 2023). In TCM research, multiple nanotechnology platforms, including exosomes, carbon dots (CDs), nanogels, polymeric micelles, self-microemulsifying drug delivery systems, and liposomes, have been widely employed for the development of novel TCM therapeutics, giving rise to a range of biological activities (Figure 1) (Yunshu et al., 2024). A nanodrug delivery system is a sub-particulate carrier system operating at the nanoscale, encompassing nanoparticles (NPs), nanoliposomes, solid lipid nanoparticles (SLNs), nano-micelles, nanoemulsions, nanocapsules, and nanogels (Batool et al., 2023; Yang H. et al., 2023; Movahedpour et al., 2023). These systems can encapsulate insoluble drugs, lipophilic compounds, protein-based therapeutics, nucleic acid drugs, and unstable or easily inactivated agents (Li L. et al., 2023; Zhang et al., 2025a; Nie and Chen, 2020). By improving TCM solubility, prolonging *in vivo* circulation time, and minimizing off-target cytotoxicity, nano-formulations may reduce the required therapeutic dose. They can also enhance biomembrane permeability and alter biodistribution, potentially enabling reinterpretation of TCM principles at the cellular, subcellular, and molecular levels.

### 3.1 Nanoemulsions

Nanoemulsions are transparent or semi-transparent, low-viscosity, isotropic, and thermodynamically stable nanoscale oil–water systems that form spontaneously when the aqueous phase, oil phase, surfactant, and cosurfactant are combined in appropriate proportions (Zhong et al., 2024). Owing to their high solubilization capacity, optical transparency, excellent thermodynamic stability, rapid diffusion, high absorption rate, and ease of preparation, nanoemulsions are considered promising alternative drug-delivery systems (Madhavi and Battu, 2024). 1,8-Cineole (CIN) has been shown to reduce atherosclerotic lesion areas by lowering blood lipid parameters and inhibiting the expression of inflammatory factors and proteins, thereby mitigating vascular tissue damage (Jiang et al., 2023). To enhance the therapeutic efficacy of CIN against AS, Chen et al. developed an oral nanoemulsion stabilized by a polysaccharide–protein complex using microjet and ultraviolet irradiation technology (Chen et al., 2023b). This nanoemulsion employed a dextran–bovine serum albumin/fish albumin complex as an emulsifier and incorporated vitamin B12 as a ligand to facilitate intestinal transport. *In vivo* evaluation in an atherosclerotic mouse model demonstrated significant anti-atherosclerotic efficacy. Moreover, this formulation improved both the *ex vivo* and *in vivo* stability of CIN, prolonged gastrointestinal retention, enhanced penetration of the mucus layer and intestinal epithelial cells, and increased oral bioavailability and plaque deposition. Collectively, these findings provide insights for the development of oral formulations containing essential oils.

### 3.2 Nanoliposomes

The term “liposome” dates back to the 1960s and was first used to describe the spontaneous formation of phospholipid bilayer vesicles in aqueous environments (Wang et al., 2024). Liposomes comprise one or more lipid bilayers surrounding an aqueous core, with diameters typically ranging from 20 to 1,000 nm. Their structure features a hydrophobic region formed by aggregation of phospholipid or sphingolipid tails and a hydrophilic region defined by the exposed head groups. Due to their structural similarity to cell membranes, liposomes can fuse with biological membranes to release encapsulated contents intracellularly and modulate cellular functions, making them well suited for targeting the intracellular compartment. This capability enhances drug selectivity for specific cells while minimizing uptake by non-target phagocytic cells (Peng et al., 2023).

Lipid nanoparticles (LNPs), also referred to as nanoliposomes, emerged in the pharmaceutical industry as an optimized variant of conventional liposomes. Beyond differences in particle size, LNPs exhibit distinct compositional and structural characteristics; for example, they typically contain a substantially higher proportion of cholesterol than traditional liposomes. Moreover, whereas conventional liposomes possess an internal hydrophilic cavity, LNPs often adopt a multilayered core structure, a feature influenced by electrostatic interactions between the encapsulated cargo (e.g., nucleic acids) and phospholipid head groups (Eygeris et al., 2022). Conventional liposomes are widely employed for the delivery of small-molecule drugs (Luo D. H. et al., 2023), whereas LNPs are predominantly utilized for nucleic acid therapeutics, including mRNA and siRNA (Golubovskaya et al., 2023). With continued advances in LNP preparation and assembly technologies, natural metabolites derived from TCM are increasingly explored as key components of LNP-based drug-delivery systems (Chatterjee et al., 2023). In recent years, the application of nanoliposomes in AS research has gained considerable momentum. Berberine (BBR) promotes autophagy in peritoneal macrophages and facilitates the nuclear translocation and deacetylation of transcription factor EB through activation of the SIRT1 protein within the NAD+ synthesis pathway, a regulatory mechanism that may represent a novel therapeutic strategy for AS. Additional studies have identified BBR as one of the most promising natural products for regulating lipid and glucose metabolism. Specifically, BBR stimulates KLF16 expression, thereby activating PPARα signaling and enhancing the interaction between KLF16 and PPARα, which alleviates AS symptoms in diabetic patients (Man et al., 2022). To overcome the bioavailability limitations of conventional BBR formulations, Du et al. developed BBR-loaded proliposomes as solid templates for preparing high-dose liposomes (Duong et al., 2022). This strategy significantly enhanced the oral bioavailability and therapeutic efficacy of BBR. The authors further evaluated the pharmacokinetics and cholesterol-lowering effects of reconstituted BBR liposomes in male Wistar rats and mice. The oral bioavailability of liposomal BBR was markedly higher than that of BBR administered as an oral suspension. This work addresses the longstanding limitation of poor bioavailability associated with traditional BBR formulations and provides a scientific foundation for developing oral liposomal therapies to treat hyperlipidemia and reduce AS risk (Kim et al., 2022; Dad et al., 2021; Dilimulati et al., 2025).

### 3.3 Nanogels

Nanogels are three-dimensional crosslinked polymer networks with diameters of < 200 nm and represent a nanoscale subclass of hydrogels (Yang X. et al., 2023). Relative to larger gel systems, nanogels offer several advantages, including enhanced cellular uptake, the ability to cross the blood–brain barrier for brain-targeted drug delivery, and high drug-loading efficiency. These properties position them as versatile carriers for promoting transdermal drug absorption, achieving controlled release, and enabling targeted delivery. When integrated with TCM components to form TCM nanogels, their therapeutic potential is substantially amplified. Owing to their high loading capacity and tunable release kinetics, nanogels are well suited for targeting vascular pathologies. For example, shikonin-loaded nanogels suppress IL-1β release by inhibiting NLRP3 inflammasome activation in macrophages, thereby attenuating the inflammatory cascade in AS (Li X. et al., 2023). Quercetin (QCT), a flavonoid widely distributed in the plant kingdom, is well recognized for its potent antioxidant and anti-inflammatory properties. An inhalable QCT–alginate nanogel (QUNG) has been developed to significantly enhance the solubility and oral bioavailability of QCT. When administered through ultrasonic nebulization, QUNG markedly reverses oxidative stress–induced damage in acute lung injury (ALI) rat models and downregulates the expression of pro-inflammatory cytokines. Moreover, by alleviating pulmonary oxidative stress through nebulized delivery, the QCT–alginate nanogel indirectly ameliorates pulmonary hypertension (Chen R. et al., 2022). Collectively, these examples underscore the potential of nanogel-based strategies to enhance the therapeutic efficacy of TCM-derived compounds in cardiovascular and inflammatory diseases.

### 3.4 Micelles

Micelles are thermodynamically stable colloidal aggregates with an ordered structure that form through surfactant self-assembly in aqueous solution upon reaching a critical concentration (Yan, 2024). When amphiphilic block copolymers undergo similar self-assembly, the resulting thermodynamically stable colloidal solutions are termed polymeric micelles (Jianqing, 2023). Owing to their minute particle size, hydrophobic water-free core, protective outer shell, solubilization capacity, and low toxicity, polymeric micelles have emerged as one of the most important platforms in contemporary drug-delivery systems (Ghezzi et al., 2021).

Among these, polyethylene glycol (PEG)-based micelles have been extensively investigated for AS therapy due to their structural simplicity and modifiable lipid tails. Given that inflammatory cascades and oxidative stress are key drivers of AS initiation and progression, PEG-based micelles have been employed to deliver anti-inflammatory agents specifically to plaques, thereby mitigating local inflammation and reducing oxidative stress. For example, Shuai et al. developed PEG-block-poly (propylene sulfide) (PEG-b-PPS) micelles that respond to the oxidative microenvironment and enable targeted delivery of andrographolide (Andro) (Wu et al., 2018). Andro, a labdane diterpenoid with potent anti-inflammatory activity, has faced clinical limitations due to its poor aqueous solubility. By leveraging the intrinsic ROS-responsive properties of PEG-b-PPS micelles, Andro-loaded formulations achieved sustained release within plaques, effectively suppressing inflammatory responses and alleviating oxidative stress *in vivo*. This study highlights the potential of ROS-responsive micelles for targeted anti-inflammatory therapy in AS.

Similarly, Scott et al. used ROS-responsive PEG-b-PSS micelles to deliver celastrol, a hydrophobic inhibitor that downregulates receptors involved in the activation of the NF-κB pathway, a critical mediator of AS pathogenesis (Allen et al., 2019). Notably, PEG-b-PSS micelles demonstrated high encapsulation efficiency for celastrol and produced significant inhibition of NF-κB signaling in RAW264.7 cells at a substantially lower effective concentration than free celastrol. *In vivo* studies further revealed that Ldlr^−/−^ mice treated with low-dose celastrol-loaded micelles exhibited significantly reduced plaque areas compared with animals receiving blank micelles or an equivalent dose of free celastrol. In addition, celastrol-loaded micelles decreased the abundance of pro-inflammatory immune cells, including neutrophils and monocytes. These findings collectively demonstrate that PEG-b-PSS micelles can markedly enhance the therapeutic efficacy of hydrophobic agents, positioning them as promising platforms for addressing localized inflammation in AS plaques.

Beyond PEG-b-PSS micelles, alternative micellar systems have also been developed for inflammation-targeted AS therapy. For example, micelles composed of d-α-tocopherol PEG succinate and loaded with the botanical drug BBR inhibited inflammation in high-fat diet-fed ApoE^−/−^ mice. This effect was attributed to disruption of macrophage–adipocyte crosstalk and downregulation of key inflammation-associated genes (Ma et al., 2020).

### 3.5 Carbon dots (CDs)

Carbon dots (CDs) are carbon-based NPs with a size of < 10 nm, possessing various functional groups on their surface. They can be applied in various fields, including biomedicine and energy. In a comprehensive review published in *Nature Nanotechnology*, Đorđević et al. systematically described how CD properties can be tailored for specific applications using chemical strategies and synthesis methods (de Boëver et al., 2022). Following high-temperature carbonization of TCM, “TCM-derived carbonaceous nanoparticles” with a particle size of < 10 nm are generated, also known as “TCM-derived carbon quantum dots,” representing an emerging class of carbon-based nanomaterials. CDs are zero-dimensional carbon nanomaterials with significant fluorescent properties, consisting of ultrafine, dispersed, quasi-spherical carbon NPs with diameters < 10 nm (Garcia-Millan et al., 2022). Depending on the carbon source, CDs can be classified as carbon quantum dots (CQDs), graphene quantum dots (GQDs), carbonized polymer dots (CPDs), or carbon nanodots (CNDs) (Mohammadi et al., 2022). Owing to their excellent optical properties, water solubility, low toxicity, and biocompatibility, CDs have been widely explored for pharmaceutical applications, including medical imaging, photothermal therapy, and anti-tumor therapy. CDs prepared from ***broccoli*** water extract (BWE) exhibit prominent antioxidant properties, effectively scavenging reactive oxygen species (ROS) in A549 cells, 293T cells, and zebrafish, and alleviating lipopolysaccharide (LPS)-induced inflammation in zebrafish (Deng et al., 2023). These effects are attributed to direct reactions between BWE-derived CDs (BWE-CDs) and free radicals, leading to modulation of nitric oxide levels and upregulation of superoxide dismutase and glutathione peroxidase-4 expression. Given the close link between oxidative stress and inflammatory responses, enhancing anti-inflammatory activity via antioxidation (ROS scavenging) is also a functional characteristic of TCM-based nanogels.

### 3.6 Solid lipid NPs

Solid lipid nanoparticles (SLNs) are a submicron drug-delivery system developed in the early 1990s. They are solid colloidal carriers with particle sizes of 10–1,000 nm that use solid natural or synthetic lipids (such as lecithin and triglycerides) as matrices, in which drugs are encapsulated or embedded within lipid cores. SLNs are also described as lipid-based NP systems with low toxicity, good biocompatibility, and biodegradability, in which drugs can be adsorbed to or encapsulated within the lipid matrix. Numerous studies have investigated TCM-based nano-formulations for the treatment of coronary heart disease associated with AS. For example, colchicine, initially extracted from *Colchicum autumnale* (Liliaceae), is an anti-inflammatory drug.

**FIGURE 2:**
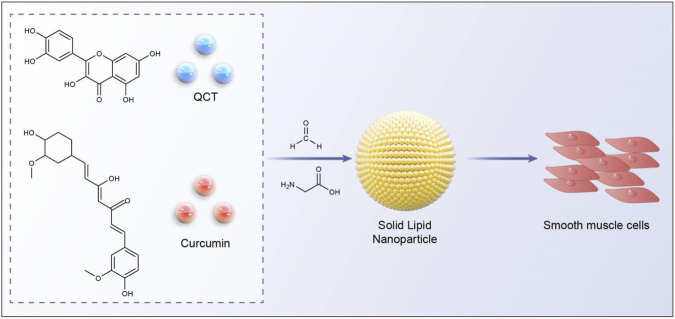
Synthesis process of nano-quercetin (N-QCT) encapsulated within SLNs. QCT and BBR interact with molecules containing carbamate structures to form solid lipid nanoparticles Solid Lipid Nanoparticle (SLNs). Subsequently, these solid lipid nanoparticles act on smooth muscle cells.

Masoumeh Shamsi et al. reported that AS remains a major contributor to death worldwide (Shamsi et al., 2024), highlighting the urgent need for innovative therapies that target chronic inflammation (Figure 2). They further noted that ***QCT and curcumin***, two naturally occurring metabolites, have potential therapeutic benefits in cardiovascular diseases. This study examined the synthesis of nano-quercetin (N-QCT) encapsulated in SLNs and evaluated the synergistic cardioprotective effects of N-QCT and curcumin in human vascular smooth muscle cells (VSMCs). The potential molecular mechanisms were investigated, with particular emphasis on TGF-β signaling in VSMCs. VSMCs (including TGF-β-stimulated VSMCs) were treated with N-QCT, curcumin, or their combination. An MTT assay was performed to evaluate cytotoxicity and the obtained data for curcumin and QCT across different concentrations were used to calculate the combination index (CI). CI analysis was performed to quantify synergy or antagonism. In addition, following TGF-β stimulation, antioxidant enzyme activity, nuclear factor erythroid 2-related factor (Nrf2) mRNA expression, ROS production, NADPH oxidase (NOX) expression, and extracellular signal-regulated kinase (Erk) 1/2 phosphorylation were measured in the treated VSMCs. N-QCT and curcumin significantly modulated Nrf2 mRNA expression and upregulated downstream antioxidant enzymes, including HO-1, GPx, and SOD1. Combined treatment further enhanced Nrf2 protein expression and regulated Erk1/2 phosphorylation. Notably, this combination exhibited synergistic cardioprotective effects, characterized by reduced ROS production and decreased Erk1/2 phosphorylation through the TGF-β/NOX/Erk1/2 and ROS/Nrf2 signaling pathways. The findings indicate that QCT and curcumin encapsulated in SLNs synergistically reduce oxidative stress and inflammation in TGF-β-stimulated VSMCs by inhibiting ROS/Erk1/2 signaling and activating Nrf2 and antioxidant enzymes. Together, these natural metabolites may represent a promising therapeutic strategy to alleviate inflammatory processes associated with AS.

### 3.7 Polymeric NPs

NPs are colloidal particles typically fabricated from biocompatible and biodegradable polymers. Owing to their ease of modification for encapsulating therapeutic agents, their ability to target specific sites, and their potential to release cargo upon specific biological or external stimuli, polymeric NPs have attracted considerable attention for biomedical applications. To date, among polymeric NPs, apart from polymeric micelles and dendrimers discussed in the following sections, the most extensively studied platforms for AS treatment are based on hyaluronic acid (HA) and poly (lactic-co-glycolic acid) (PLGA) (Aili et al., 2024). HA is a linear biopolymer, a polysaccharide and hydrated polyanionic macromolecule composed of alternating units of N-acetylglucosamine and β-glucuronic acid, and is a major component of the extracellular matrix (ECM). Owing to its biodegradability, low toxicity, non-immunogenicity, lack of inflammatory responses, and ability to bind stabilin-2 and cluster of differentiation 44 (CD44), HA-based NPs (HANPs) have been widely investigated for targeted delivery of anticancer agents. The use of HANPs as therapeutic delivery systems for AS treatment remains at an early stage (Nacu et al., 2024). However, macrophage polarization is well recognized as a key determinant of AS progression and regression; accordingly, numerous studies have examined whether HANPs loaded with different cargoes can reprogram macrophage polarization (Zhang et al., 2024). HANPs may also serve as alternatives to HA-ATNPs for future AS treatment. For example, in 2018, Santos et al. reported that HA-PLANPs containing ***curcumin*** (Cur), HA–polyethyleneimine (PEI) NPs containing microRNA-233 (miR-233), and HA-PEI carrying plasmid DNA could modulate macrophage polarity (Shahbazi et al., 2018).

### 3.8 PLGA NPs

PLGA is an FDA-approved, biodegradable, and biocompatible copolymer composed of lactic acid and glycolic acid. Owing to its excellent biocompatibility and biodegradability, PLGA has been widely used to fabricate NPs for drug delivery in cancer therapy (Xiao et al., 2025). In addition, PLGA NP surfaces can be functionalized with ligands to enable targeted delivery to specific disease sites. In AS research, several studies have focused on using PLGA NPs to deliver therapeutic agents, primarily conventional drugs and pro-resolving mediators, to modulate molecular pathways relevant to disease. Yang et al. evaluated PLGA NPs co-delivering vascular endothelial growth factor (VEGF) plasmids and ***paclitaxel*** (PTX) to prevent restenosis. Specifically, the NP consisted of a PLGA core encapsulating PTX, with a VEGF plasmid as the outer layer. Local administration of VEGF–PTX–PLGA NPs promoted reendothelialization in AS plaques *in vivo* and inhibited smooth muscle cell (SMC) growth (Xu et al., 2023). Beyond PLGA NPs and HANPs, significant progress has been made recently in the development of polymeric NPs composed of other types of polymers for AS treatment. For example, one study reported that polymeric nanoparticles assembled from chitosan (CS) and polyaspartic acid (PAA) can deliver ***EGCG***, which reduces the area of rabbit aorta occupied by lipid deposits (Shao et al., 2025). Furthermore, Moghe et al. developed antioxidant polymeric nanoparticles to regulate macrophage adipogenesis and ROS production within AS plaques (Kim et al., 2023). These NPs consist of a ferulic acid–based polyester core and a shell modified with amphiphilic molecules that target scavenger receptors. Because ***ferulic acid*** is a natural antioxidant reported to reduce macrophage adipogenesis, these ferulic acid–based NPs downregulated CD36 and LOX-1 expression in macrophages and inhibited macrophage uptake of oxidized low-density lipoprotein (ox-LDL).

**FIGURE 3:**
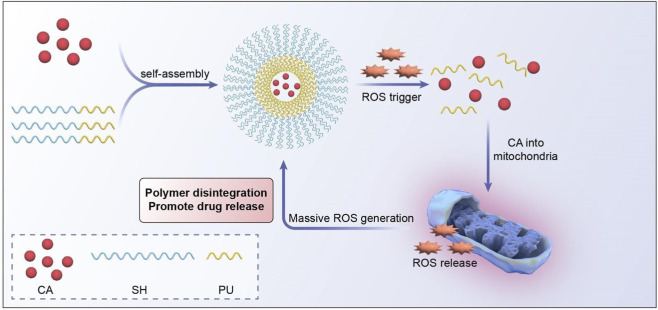
Schematic of RGCP@PU NPs redesigned according to reference (Liu et al., 2025a). Self-assembly of RGCP@PU NPs (a nanoparticle system) and the ROS-related process of responsive drug release: First, components such as CA, SH, and PU self-assemble to form RGCP@PU NPs; when high levels of ROS are generated, ROS promotes polymer disintegration, thereby facilitating drug release. In addition, upon ROS triggering, CA can enter mitochondria, which also release ROS and participate in related biological processes.

Yunxiao designed a novel ROS-responsive CA polymer, and developed ***polyurethane*** (PU)-loaded nanoparticles, termed RGCP@PU NPs (Figure 3) (Liu et al., 2025a). These nanoparticles are designed to respond to elevated ROS levels in AS lesions and to release PU for AS treatment. The study demonstrated that RGCP@PU nanoparticles exhibit favorable morphological and physiological stability. They not only significantly reduced oxidative stress and inflammation *in vitro*, but also exhibited excellent biocompatibility *in vivo* while effectively inhibiting AS progression. Collectively, these findings highlight RGCP@PU NPs as a promising therapeutic strategy for AS.

## 4 Biological/bionic nanocarriers

AS is a primary pathological basis of cardiovascular disease and has a complex pathogenesis involving inflammation, dysregulated lipid metabolism, and oxidative stress. TCM nano-formulations have shown promising potential for AS treatment. Their mechanisms can be summarized as follows: by altering *in vivo* drug distribution (pharmacokinetics), nano-formulations enable targeted delivery to diseased tissues and improve the therapeutic index (efficacy-to-toxicity ratio) (Duivenvoorden et al., 2019). The enhanced permeability and retention (EPR) effect is a major mechanism underlying passive targeting of nano-formulations. Building on the EPR effect, more precise active targeting can be achieved by modifying NP surfaces with targeting ligands (e.g., antibodies, peptides, aptamers, and small molecules). In addition, encapsulating NPs in lesion-adaptive matrices (e.g., polymers, antibodies, proteins, aptamers, or peptides) can increase preferential contact with AS lesions and facilitate penetration into the leaky vasculature at these sites (Moghimi et al., 2005). TCM nano-formulations primarily leverage these principles by incorporating active TCM metabolites into nanodelivery systems to create integrated therapeutic platforms. Compared with conventional NPs, bionic biological nanomaterials (bNMs) can provide prolonged circulation, improve immune evasion, and enhance therapeutic efficacy (Tao et al., 2023). For imaging-based detection of AS plaques (particularly vulnerable plaques), bNMs can support non-invasive diagnosis while also offering substantial drug-loading capacity, enabling accurate delivery of diagnostic molecules to lesion sites and thereby providing dual functionality. Multiple studies have reported bNM-based loading and delivery of active TCM components.

### 4.1 Natural biomolecules camouflage nanoparticles as biocompatible components

Natural biomolecules can bind to, or self-assemble into, nanocarriers to endow bNMs with targeting and stealth capabilities. Natural-biomolecule-derived carriers (NBCs) deliver drugs to AS lesions by exploiting the binding of endogenous ligands to cellular receptors or by using natural targeting macromolecules such as HDL and human serum albumin (HSA). These biomimetic carriers can replicate interactions between endogenous molecules and cells, as well as cell–cell interactions (He et al., 2025). Atherosclerotic bNM carriers containing HDL lipoproteins are circulating, heterogeneous natural nanoparticles produced by the liver and intestines with inherent stability and low immunogenicity (Kontush and Chapman, 2010). In particular, HDL has a diameter of < 30 nm, enabling plaque penetration and cholesterol efflux from lipid-rich foam macrophages. HDL also confers additional benefits, including anti-inflammatory and antioxidant effects that support cardiovascular protection (Gill et al., 2021).

***Tanshinone IIA***, an active component of Salvia miltiorrhiza (Danshen), is widely studied in AS research. Zhang Wenli et al. developed a drug-loaded reconstituted HDL (rHDL) system with dual functionality: targeting drugs to atherosclerotic plaques while also providing therapeutic effects attributable to the carrier itself (Zhang et al., 2011). The authors selected tanshinone IIA (TA), a well-known TCM compound used for treating AS, as a model drug. From the perspective of developing a novel delivery system, they constructed and characterized TA-loaded rHDL (TA-rHDL) with different structures, assessing particle size, encapsulation efficiency, morphology, and apo–lipid interactions. TA-rHDL preserved the discoidal and spherical morphologies of natural HDL and exhibited preferential targeting to foam cells. These findings provide useful guidance for designing rHDL systems for delivery of lipophilic cardiovascular drugs. The authors also proposed further studies to evaluate TA-rHDL size-dependent effects, *in vivo* plaque targeting, and pharmacokinetics/pharmacodynamics.

### 4.2 Atherosclerotic bNM carriers containing platelets

Fontana et al. developed a biomimetic nanocarrier for delivering the anti-inflammatory compound ***curcumin***, in the context of AS therapy (Fontana et al., 2024). The team loaded curcumin into lignin, a wood-derived biopolymer, modified the particle surface with a TA–iron complex, and further performed camouflage treatment using platelet membrane fragments to confer stealth properties. The obtained NPs exhibited a uniform particle size distribution and a negative surface charge, showing high stability during storage in ultrapure water at +4 °C, remaining stable even after vigorous mixing in cell culture medium at temperatures up to 37 °C. Drug release was ∼75% within the first 2 h at pH 7.4, whereas ∼50% was released at pH 5.5, followed by a more sustained release over the subsequent 24 h. These NPs demonstrated good cytocompatibility with endothelial cells and smooth muscle cells, whereas curcumin exhibited toxicity in specific immune cell models at high concentrations. Surface modification significantly enhanced NP interactions with endothelial cells; this effect was similarly observed in macrophage-like cells under both steady-state and lipopolysaccharide (LPS)-induced inflammatory conditions.

In addition, ***notoginsenoside R1***, an important active component of the TCM Panax notoginseng, can dissipate blood stasis and stop bleeding, as well as exert therapeutic effects in cardiovascular diseases (Lin et al., 2025). However, its therapeutic efficacy is limited by low oral bioavailability. To address this limitation, researchers conjugated a CD11b antibody to mesoporous silica nanoparticles (MSNs) loaded with notoginsenoside R1, generating MSN-NGR1-CD11b NPs. By exploiting the infiltration and accumulation of neutrophils and monocytes at sites of early myocardial ischemic injury, this strategy markedly enhanced delivery of notoginsenoside R1 to injured myocardium. These NPs improved cardiac function, reduced cardiomyocyte apoptosis, induced macrophage polarization toward the M2 phenotype, promoted angiogenesis, and attenuated inflammation at the infarct site (Meng et al., 2019).

### 4.3 Cell-derived carriers camouflage NPs as cell mimics

In recent years, cell-derived carriers have been widely used as nanodrug delivery systems for AS-related integrated diagnosis and therapy. These carriers primarily include extracellular vesicles (EVs) and cell membrane (CM)-coated NPs.

#### 4.3.1 NPs encapsulated in extracellular vesicles

***Ginger***-derived exosome-like nanovesicles have a diameter of 50–200 nm and contain bioactive components, including lipids, proteins (e.g., enzymes, actin, and channel proteins/transporters), microRNAs, and bioactive metabolites (e.g., 6-gingerol, 8-gingerol, 10-gingerol, and 6-shogaol). Gingerols and shogaols exhibit anti-inflammatory activity; for example, 6-gingerol inhibits nuclear factor κB (NF-κB) activation and protein kinase C (PKC) translocation, thereby suppressing cytokine production and T cell activation (Zhu and He, 2023). Garlic-derived exosome-like nanovesicles (GENs) contain 26 lipid species, 61 proteins, and 127 known microRNAs (miRNAs). GENs significantly downregulate the expression of Toll-like receptor 4 (TLR4), myeloid differentiation primary response gene 88 (MyD88), and NF-κB and reduce dextran sulfate sodium (DSS)-induced secretion of pro-inflammatory cytokines; Han-miR3630-5p in GENs binds the 3′ untranslated region (3′ UTR) of TLR4, thereby inhibiting TLR4 expression (Zhu et al., 2023).

Yunchan et al. (Sui et al., 2025) investigated the effects of ***mulberry***-derived extracellular vesicle-like nanoparticles (MFEVLPs) on dyslipidemia and AS progression. MFEVLPs were isolated by ultracentrifugation and purified by sucrose density-gradient centrifugation, and were characterized using transmission electron microscopy (TEM) and nanoparticle tracking analysis (NTA). Small RNA sequencing (small RNA-seq) was performed to profile miRNAs. Free fatty acid (FFA)-stimulated HepG2 cells and high-fat diet-fed ApoE^−/−^ mice, including the model, MFEVLP treatment, and atorvastatin treatment groups, were used to investigate the effects of MFEVLPs on dyslipidemia and AS progression. miRNA mimics and luciferase assays were used to analyze the interactions between MFEVLP-derived miRNAs and target genes. MFEVLPs reduced plaque area and enhanced plaque stability in ApoE^−/−^ mice as well as inhibited hepatic lipid synthesis and decreased hepatic and serum lipid levels *in vitro* and *in vivo*. MFEVLPs contained abundant miRNAs, including 20 miRNAs predicted to target genes involved in adipogenesis (SREBP1, FAS, and ACC) and cholesterol biosynthesis (HMGCR).

**FIGURE 4:**
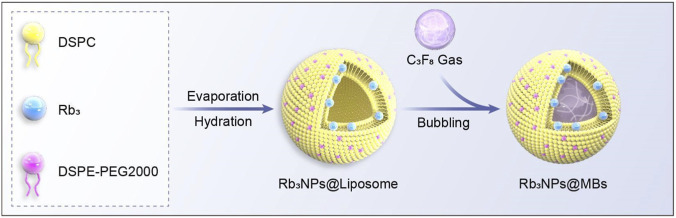
Representation of Rb3NPs@MBs combined with UTMD for targeted therapy of atherosclerosis, redesigned according to reference (Zhong et al., 2025). Schematic of the preparation process from liposomes (Rb_2_/NPs@Liposome) to microbubbles (Rb_2_/NPs@MBs). First, DSPC, Rb_2_, and DSPE-PEG2000 are used as raw materials to form Rb_2_/NPs@Liposome via evaporation–hydration; C_6_F_14_ gas is then introduced (gas bubbling) to convert Rb_2_/NPs@Liposome into Rb_2_/NPs@MBs (microbubbles).

Chunting et al. developed a ***saponin***-encapsulated ultrasound microbubble system (Rb3NPs@MBs) combined with ultrasound-targeted microbubble destruction (UTMD) to enrich drug delivery to target regions in AS mice under ultrasound guidance (Figure 4) (Zhong et al., 2025). Using this delivery system, they demonstrated that Rb3NPs@MBs reduced aortic plaques in AS mice and explored the underlying mechanisms. This microbubble system effectively encapsulated saponin Rb3 nanoparticles and, via UTMD, promoted targeted accumulation in the aortic arch. Rb3NPs@MBs subsequently reduced oxidative stress, attenuated endothelial cell apoptosis and foam cell formation, and ultimately inhibited plaque development at lesion sites.

#### 4.3.2 Cell membrane-coated NPs

**FIGURE 5:**
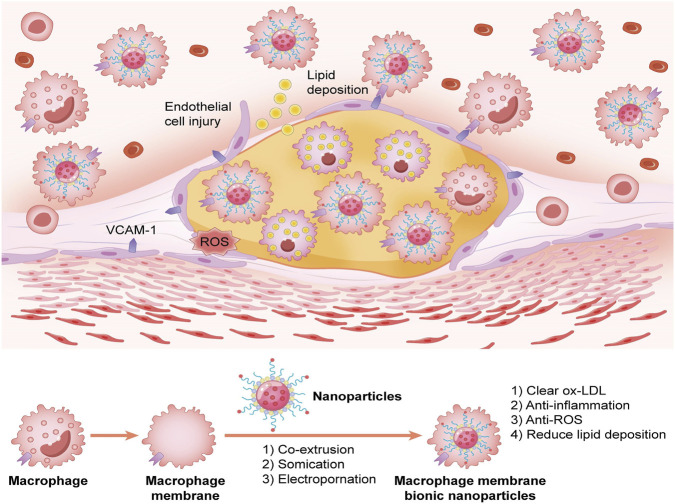
Schematic of targeted therapy for atherosclerosis using functionalized macrophage membrane biomimetic nanoparticles, drawn according to reference (Zhang et al., 2025a). The figure illustrates how nanoparticles (Nanoparticles) address macrophage (Macrophage)-related processes and ameliorate endothelial cell injury (Endothelial cell injury) and lipid deposition (Lipid deposition), with involvement of factors such as ROS and vascular cell adhesion molecule-1 (VCAM-1). Nanoparticles can be functionalized on the macrophage membrane via co-extrusion (Co-extrusion), incubation (Incubation), or electroporation (Electroporation) to promote oxidized low-density lipoprotein clearance (Clear ox-LDL), exert anti-inflammatory effects (Anti-inflammation), scavenge ROS, and reduce lipid deposition (Reduce lipid deposition).

In particle surface modification, a top-down biomimetic strategy was investigated by coating biodegradable polymer NPs with natural cell membranes (CMs) to enable long-acting drug delivery and targeted accumulation at lesions (as shown in Figure 5).

**FIGURE 6:**
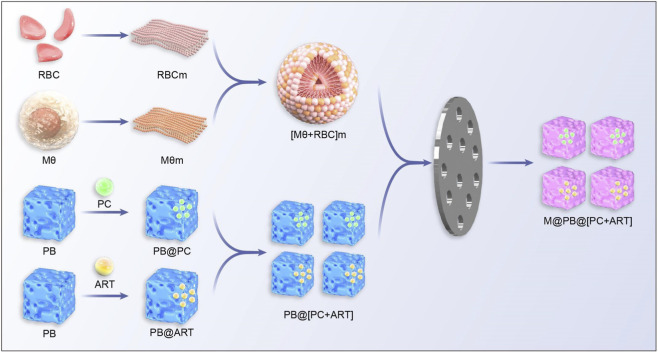
M@PB@(PC+ART) NPs redrawn according to reference (Zhou et al., 2022). The schematic illustrates a cell engineering-related preparation workflow in which red blood cells (RBCs), macrophages (Mac), pancreatic cancer cells (PancPC), and prostate cancer cells (PrCaRT) are employed to construct cell structures, including a Mac@RBC membrane (Mac@RBC Tm), PancPC@CAR-T, and PrCaRT@CAR-T. These designs ultimately yield Me@PancPC@CAR-T and subsequent products such as HLA-R@Me@PancPC@CAR-T cell products, which may be used in cell therapy-related research and applications.

Hongyan et al. constructed a biomimetic membrane-coated drug-delivery nanocomposite based on Prussian blue nanoparticles (PB NPs) for AS treatment (Figure 6) (Zhou et al., 2022). First, PB NPs, which have high drug-loading capacity, were used to load ***artemisinin (ART)*** and ***proanthocyanidins*** (PC). Next, a fused macrophage and red blood cell membrane ([Mø+RBC]m) was used to camouflage PB@(PC+ART) NPs, yielding M@PB@(PC+ART) NPs, which enhanced NP recruitment to plaque regions and prolonged circulation time. Finally, HA-PEG2000-DSPE (hyaluronic acid–polyethylene glycol 2000–distearoylphosphatidylethanolamine) was inserted into [Mø+RBC]m to enable targeting of inflammatory macrophages within plaques. This ART/PC co-loaded nanocomposite was therefore designed to achieve controlled drug release in inflammatory macrophages and to alleviate AS by inhibiting the RONS/NF-κB/NLRP3 pathway, activating the AMPK/mTOR/autophagy pathway, and concurrently regulating lipid influx and cholesterol efflux.

**FIGURE 7:**
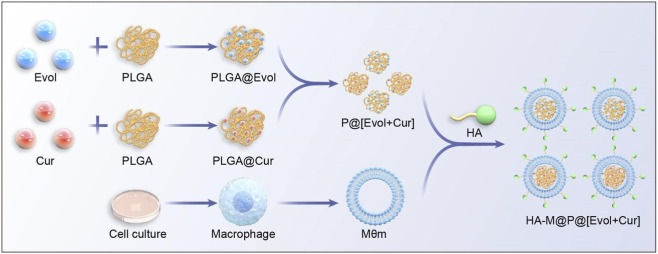
Preparation principle of HA-M@P@(Evol+Cur) nanoparticles and the corresponding strategy for atherosclerosis treatment, redrawn according to reference (Liu et al., 2025b). Resveratrol (Res) and curcumin (Cur) are separately combined with PLGA to form PLGA@Res and PLGA@Cur, while macrophages (Mac) are obtained through cell culture. PLGA@Res and PLGA@Cur are then incubated with macrophages to form PLGA@Res/Cur@Mac, which is subsequently processed to obtain macrophage vesicles loaded with the relevant cargos (Mac@PLGA@Res/Cur).

Yi et al. developed a nanodrug delivery system, HA-M@P@(Evol+Cur) NPs, modified with fused macrophage membrane (Møm) camouflage and hyaluronic acid (HA) (Figure 7) (Liu et al., 2025b). The Møm coating enables immune evasion, whereas HA interacts with CD44 receptors highly expressed on activated macrophages, promoting selective enrichment at plaque sites. By integrating passive and active targeting, this delivery system improves *in vivo* drug retention. In this system, Evol NPs can directly reduce cholesterol deposits in AS plaques and liver tissue and provide dual protective effects, while maintaining the anti-inflammatory activity of ***curcumin*** (Cur) to delay AS progression. The performance of the nanodelivery system was validated *in vitro* and *in vivo*; in apolipoprotein E-deficient (ApoE^−/−^) mice, NPs showed targeted accumulation in AS plaques and liver tissue. Mechanistic studies further indicated that NPs promoted lipid efflux.

**FIGURE 8:**
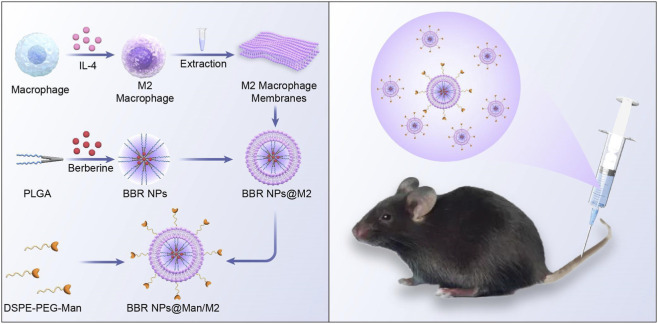
Construction process of a macrophage-based berberine nanoparticle (BBR NPs) delivery system, redrawn according to reference (Wu et al., 2024). Macrophages are first induced by IL-4 to generate M2 macrophages, and their membranes are extracted. In parallel, BBR NPs are prepared using materials including PLGA and DSPE-PEG-Man. The BBR NPs are then combined with the M2 macrophage membrane to form BBR NPs@M2, which can be further modified with mannose to obtain BBR NPs@Man@M2 for targeted drug-delivery studies.

Haopeng et al. developed a BBR-loaded PLGA nanoparticle delivery system camouflaged with M2 macrophage membranes (BBR NPs@Man/M2) for targeted AS treatment (Figure 8) (Wu et al., 2024). Membrane camouflage combined with mannose (Man) modification confers inflammation-oriented chemotaxis, enabling preferential targeting of AS plaque regions. After accumulation at the lesion site, BBR NPs@Man/M2 can promote polarization of M1 macrophages toward the M2 phenotype, thereby alleviating local inflammation and exerting anti-inflammatory effects. Additional studies showed that this system attenuated endothelial cell (EC) inflammatory responses, promoted EC repair and collagen secretion, and helped maintain vascular structural stability. During preparation, M2 macrophage membranes were first coated onto BBR-loaded PLGA NPs, followed by mannose modification; the physicochemical properties of the resulting biomimetic NPs were systematically characterized. Biocompatibility and biological functions were evaluated *in vitro*, and targeting capability, therapeutic efficacy, and biosafety were verified in an AS mouse model. Collectively, this delivery system provides a potential biomimetic strategy for AS treatment.

**FIGURE 9:**
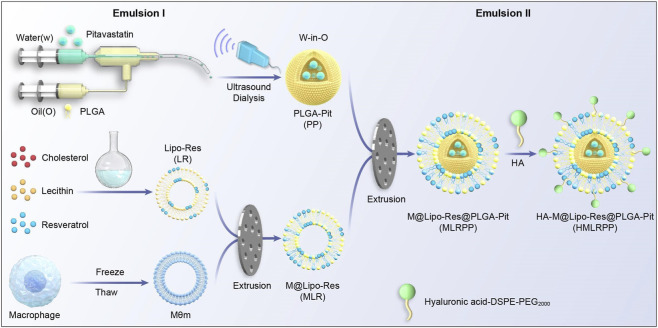
Schematic of the preparation of HMLRPP NPs, redrawn according to reference (Yang et al., 2025). First, PLGA precursor-related structures (PLGA-Pr, PP) are prepared via steps including Emulsion I, ultrasound, and dialysis. In parallel, macrophages (Macrophages, Mam) are subjected to freeze-thaw processing, combined with resveratrol (Resveratrol, Res) and pitavastatin (Pit), and extracted to obtain resveratrol-loaded macrophage vesicles (M@Lipo-Res, MLR). MLR is then combined with PLGA-Pr and other components and extracted to obtain M@Lipo-Res@PLGA-Pr (MLRPP). Finally, following Emulsion II and incorporation of hyaluronic acid (HA)-modified distearoylphosphatidylethanolamine-polyethylene glycol (HA-DSPE-PEG_2000_), the HA-modified M@Lipo-Res@PLGA-Pr (HA-MLRPP) is obtained for targeted drug delivery and related biomedical applications.

Anning et al. developed a nanomedicine, HMLRPP, comprising pitavastatin (Pit)-loaded poly (lactic-co-glycolic acid) (PLGA) NPs and ***resveratrol*** (Res)-loaded liposomes as the core, with an outer coating of macrophage membranes further modified with hyaluronic acid (HA) (Figure 9) (Yang et al., 2025). HMLRPP NPs can continuously release Pit and Res under acidic conditions and mitigate hyperhomocysteinemia (HHcy)-induced AS by inhibiting macrophage ferroptosis, attenuating inflammation, and improving disordered lipid metabolism. The study further identified several proteins, including β-hydroxybutyrate dehydrogenase 1 (BDH1), orosomucoid 1 (ORM1), and ribosomal protein S27-like (RPS27L), and several non-target metabolites, including ropinirole, desethyl ciprofloxacin, acetylenedicarboxylate, and N-acetyl-L-glutamate, as being involved in HMLRPP NP–mediated regulation of ferroptosis. Collectively, these findings indicate that HMLRPP NPs effectively modulate ferroptosis and represent a potential AS treatment strategy with high bioavailability and low side effects.

**FIGURE 10:**
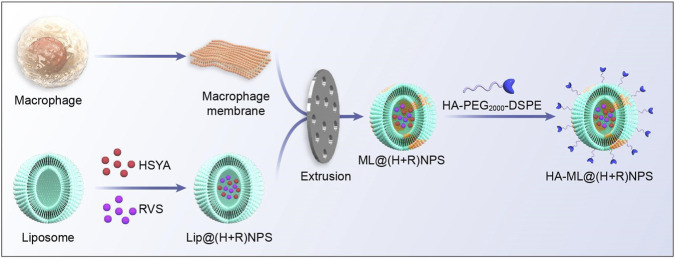
HA-ML@(H+R) nanoparticle preparation scheme, redrawn according to reference (Dong et al., 2025). The figure presents the construction of a macrophage-based nanodrug delivery system in which macrophages are combined with liposomes and subsequently treated with HISTA and LPS to sequentially form M@(H + R) NPs; these are then modified with HA-PEG_2000_-OH to obtain HA-M@(H + R) NPs for constructing a targeted drug delivery system.

Xiaoyan et al. developed a plaque/macrophage dual-targeted programmed AS management strategy that simultaneously regulates lipid metabolism and macrophage phenotype using biomimetic NPs derived from macrophage membranes (Dong et al., 2025). Hyaluronic acid–polyethylene glycol 2000-distearoylphosphatidylethanolamine (HA-PEG2000-DSPE), which has high affinity for the macrophage-specific surface marker CD44, was used to modify hybrid liposomes to improve the co-delivery efficiency to AS lesions (as shown in Figure 10). *In vitro*, HA-ML@(H+R) NPs reversed macrophage polarization from M1 to M2 and regulated lipid metabolism by downregulating CD36 to activate autophagy. *In vivo*, this biomimetic system effectively delivered ***hydroxysafflor yellow A*** (HSYA) and resveratrol (RV) to plaque sites, significantly enhancing plaque stability, reducing blood lipid levels, and decreasing inflammatory cytokines; these effects markedly improved homocysteine (Hcy)-induced AS in ApoE^−/−^ mice. Therefore, HA-ML@(H+R) NPs show translational potential for broader clinical application.

**FIGURE 11:**
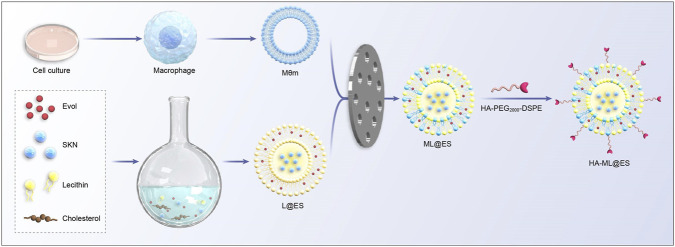
Preparation of HA-ML@ES NPs, redrawn according to reference (Zhang Q. et al., 2025). Using Evol, SKN, lecithin, cholesterol, and other raw materials, a structure containing the relevant components is prepared, which is then combined with cell membrane (Mem) to obtain L@E@S. Next, following the indicated processing step (e.g., the separation/processing step shown by the circular device in the middle), ML@E@S is obtained. Finally, introduction of hyaluronic acid-modified distearoylphosphatidylethanolamine-polyethylene glycol (HA-PEG2000-DSPE) yields HA-ML@E@S, a platform applicable to targeted drug delivery and related biomedical applications.

Zhang Qi et al. developed a dual-targeted biological liposomal nanomedicine (HA-ML@ES NPs) capable of co-loading ***shikonin*** (SKN) and evolocumab (Evol) (Figure 11) (Zhang Q. et al., 2025). HA-ML@ES NPs, modified with macrophage membranes (Mø m) and HA, prolonged drug half-life and enabled efficient co-delivery to damaged endothelial cells and inflammatory macrophages within AS plaques. SKN and Evol contributed to repairing endothelial dysfunction, inhibiting inflammation, and restoring cholesterol flux balance in macrophages, thereby supporting treatment of early-stage AS. *In vitro*, HA-ML@ES NPs simultaneously targeted dysfunctional endothelial cells and inflammatory macrophages through HA–CD44 interactions. *In vivo*, the long-circulating, plaque-accumulating HA-ML@ES NPs effectively attenuated endothelial cell dysfunction by inhibiting glycolysis and restored cholesterol flux homeostasis in macrophages by reprogramming macrophage phenotype, ultimately alleviating AS progression. Overall, these results support a dual-cell therapeutic strategy based on HA-ML@ES NPs for the treatment of early-stage AS.

#### 4.3.3 Self-assembled organic molecular pairs

In recent years, the discovery and design of self-assembled organic molecular pairs have become a rapidly developing field. Compounds with specific structural features can self-assemble into NPs to enhance antibacterial activity (Li et al., 2019; Tian et al., 2022; Huang et al., 2020), reduce toxicity (Wang et al., 2021), improve selectivity (Chen S. Y. et al., 2022), and strengthen therapeutic efficacy (Ji et al., 2022). Natural small-molecule self-assembly systems offer several advantages, including high drug-loading efficiency, good biocompatibility, controllable degradability, and synergistic pharmacological activity (Zhu et al., 2022). To date, research on self-assembly has mainly focused on physicochemical properties and *in vitro* effects, offering potential routes to overcome limitations of conventional oral drugs and nanocarrier-dependent delivery systems for AS treatment.

**FIGURE 12:**
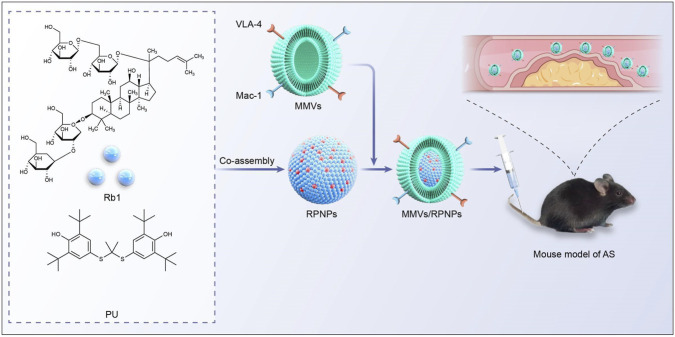
Schematic of a nanodrug delivery system based on ginsenoside Rb1 (Rb1) and PU, redrawn according to reference (Zeng et al., 2025). First, Rb1 and PU co-assemble to form Rb1/PU nanoparticles (RPNPs). Next, RPNPs are combined with macrophage membrane vesicles (MMVs) to form MMVs/RPNPs. Finally, MMVs/RPNPs are administered via injection into a mouse model of atherosclerosis, in which molecules such as VLA-4 and Mac-1 on the MMV surface may contribute to targeting for therapeutic studies of related diseases.

Jieying et al. proposed an “integration of traditional Chinese and Western medicine” strategy and developed biomimetic co-assembled NPs composed of Chinese and Western medicine components (MMVs/RPNPs) for targeted AS treatment (Figure 12) (Zeng et al., 2025). This study demonstrated for the first time that ***ginsenoside Rb1*** can co-assemble with probucol to form stable, carrier-free NPs (RPNPs) without excipients; these particles were subsequently coated with macrophage microvesicles (MMVs) to yield MMVs/RPNPs with AS-targeting capability. The resulting MMVs/RPNPs exhibited excellent intracellular ROS-scavenging capacity, inhibited pro-inflammatory cytokine secretion, and reduced lipid deposition *in vitro*. In an AS mouse model, intravenously administered MMVs/RPNPs effectively accumulated at lesion sites, significantly delayed AS progression, and promoted plaque stabilization through synergistic anti-oxidative stress, anti-inflammatory, and lipid-lowering effects. In addition, no obvious adverse reactions were observed during long-term treatment. This work provides a simple, effective, and safe nanotherapeutic strategy for AS and highlights the broad potential of “integration of traditional Chinese and Western medicine” for treating cardiovascular and cerebrovascular diseases.

**FIGURE 13:**
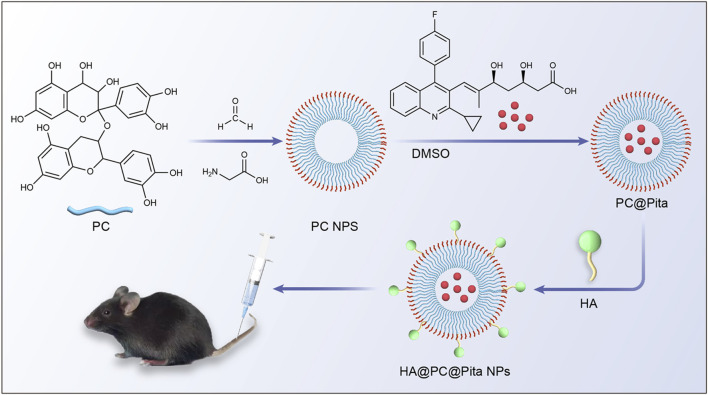
Schematic of the preparation process of a nanodrug delivery system and its application in animal experiments, redrawn according to reference (Wu Y. et al., 2025). First, PC undergoes a reaction to form PC nanoparticles (PC NPs). Subsequently, PC NPs are combined with pitavastatin (Pita) in the presence of DMSO to form PC@Pita NPs. Next, hyaluronic acid (HA) is introduced to modify PC@Pita NPs, yielding HA@PC@Pita NPs. Finally, HA@PC@Pita NPs are administered via injection into mice for related biomedical research.

Yizhou et al. constructed self-assembled ***procyanidin*** (PC) NPs for loading pitavastatin (Pita) (Figure 13) (Wu Y. et al., 2025). The designed hyaluronic acid (HA)-modified HA@PC@Pita NPs combine the advantages of Pita in restoring efferocytosis and PC in enhancing cholesterol efflux. *In vitro* experiments showed that HA@PC@Pita NPs induce M1/M2 repolarization and upregulate ERK5/Mertk expression to restore macrophage efferocytotic function. Meanwhile, HA@PC@Pita NPs significantly promote cholesterol efflux by enhancing macrophage adipophagy (selective autophagy of lipid droplets). *In vivo* studies demonstrated that, in an ApoE^−/−^ mouse model of advanced AS, HA@PC@Pita NPs reduce the necrotic core and enhance plaque stability. These findings support the potential of HA@PC@Pita NPs for the treatment of advanced AS.

## 5 Limitations and challenges of nano-loaded TCM preparations

### 5.1 Development of oral drug biomimetic targeted delivery systems

Nanoparticulate oral formulations offer significant advantages for the treatment and diagnosis of AS (Yin et al., 2020). These formulations can enable precise drug delivery through surface modification or rational design, facilitating delivery to AS plaques while reducing adverse effects on healthy tissues (Eskandani, 2025; Sun et al., 2024). By regulating NP size and architecture, controlled drug release can be achieved, ensuring that drugs are released at the optimal time and location, thereby improving therapeutic efficacy (Zhang S. et al., 2022). As an emerging strategy, oral TCM nanomedicines, which encapsulate or co-assemble active TCM metabolites (e.g., berberine, ginsenosides, resveratrol), can address key limitations, including low oral bioavailability, poor *in vivo* stability, and insufficient targeting. These delivery systems can enhance intestinal absorption of active metabolites and promote preferential accumulation at atherosclerotic lesions via functional modifications (e.g., macrophage membrane–mimetic coatings or inflammation-targeting ligands) (Luo T. et al., 2023). Proposed therapeutic mechanisms mainly include multi-pathway, synergistic actions such as inhibiting lipid deposition, alleviating oxidative stress, regulating macrophage polarization, and reducing inflammatory responses, thereby delaying plaque progression and supporting restoration of vascular homeostasis. These systems may offer low side-effect profiles and high patient compliance, providing translationally relevant opportunities for AS prevention and treatment. However, orally administered nanomedicines must still overcome biological barriers (e.g., gastric acid and the gastrointestinal mucosa), which can inactivate or degrade certain drugs and reduce the therapeutic effect. Since AS treatment often requires site-specific intervention, precise delivery to plaque regions is essential (Tang D. et al., 2021). However, oral nanoparticulate formulations still face challenges in targeted delivery, as they may distribute across multiple tissues in the body rather than acting selectively at lesion sites. In addition, long-term NP safety remains insufficiently defined, underscoring the need for rigorous evaluation of potential risks. Future oral nanoparticulate formulations may extend beyond drug delivery to integrate functions such as inflammation modulation, gene therapy, and tissue engineering for more comprehensive AS management (Yingyu et al., 2023). The development of new technologies for monitoring and tracking the *in vivo* distribution and efficacy of oral nanoparticulate formulations will further clarify mechanisms of action. Progress in this area will likely depend on continued innovation and interdisciplinary collaboration.

Zhang et al. developed an oral biomimetic targeted delivery system composed of yeast-derived microcapsules (YC) loaded with rapamycin (RAP) (Zhang et al., 2017). This system enters Peyer’s patches in the gastrointestinal tract via macrophage-mediated transcytosis and ultimately reaches lesion sites. These “Trojan horse” NPs not only reduce toxicity risks associated with non-specific distribution but also enhance the anti-AS efficacy of RAP. Similarly, when YC is used to encapsulate binarit (BIN), a selective MCP-1 inhibitor, YC can be transported by peripheral blood monocytes to AS lesions, where BIN inhibits MCP-1 secretion by activated endothelial cells (Lee, 2014). Collectively, these studies suggest that loading natural metabolites may help reduce the risk of adverse reactions.

### 5.2 Targeting the migration of other immune cells to inflammatory sites

Currently, most reported nanomaterials for the diagnosis and treatment of AS primarily target macrophages or endothelial cells. However, plaques also contain substantial numbers of T cells and B cells, as well as calcified regions. Therefore, it is necessary to develop strategies based on natural nanomaterials and modified with ligands that target these additional plaque components to improve the imaging and therapeutic efficiency of AS (Zheng et al., 2025). For example, PEGylated single-walled carbon nanotubes (PEG-SWNTs) can use circulating monocytes as shuttle carriers to target inflamed vascular sites and interact with macrophages in AS; further encapsulation of CD47-blocking small molecules enables construction of a “Trojan horse”-like NP to improve drug delivery, promote clearance of apoptotic cells within lesions, and significantly reduce *in vivo* toxicity. By leveraging the migration of immune cells to inflammatory sites, monocyte-based drug-loading systems designed for AS can specifically target damaged endothelial cells and, by loading antioxidants, effectively scavenge intracellular or exogenous ROS to delay AS progression (Chu et al., 2015). In addition, activated neutrophils can deliver NPs across the vascular barrier into lesions, thereby enhancing targeted therapeutic effects (Shan Y. et al., 2025). Neutrophils play a key role in AS development; they can be recruited to plaques and initiate innate immune responses (Meyer-Lindemann et al., 2022). By exploiting their targeting and release properties, drugs can be accurately delivered to the AS inflammatory microenvironment and achieve controlled release when neutrophils form extracellular traps.

#### 5.2.1 Development of theranostics based on natural products or biotherapeutics

A future direction may focus on developing theranostics by leveraging the therapeutic effects of natural products or biotherapeutics. It is of interest to design nanomaterials that co-deliver dual therapeutic agents to plaques and to investigate the synergistic therapeutic effects of these combined therapies. Nanomaterials, particularly NPs, have shown potential to improve imaging-based diagnosis of AS. Computed tomography (CT), magnetic resonance imaging (MRI), positron emission tomography (PET), and ultrasound are four widely used molecular imaging modalities for identifying and diagnosing plaque development in the arterial system. However, limitations including low sensitivity and limited tissue penetration have restricted their broader application compared with other imaging approaches used in cardiovascular diseases. These limitations motivate further optimization through nanomaterial-based strategies (Zhang et al., 2019). Nanomaterial-assisted molecular imaging uses exogenous contrast agents to visualize target sites during AS development and progression. Such contrast agents can be engineered to target key molecular and cellular components involved in AS pathogenesis, including ECs, smooth muscle cells (SMCs), macrophages, platelets, ECM, and cell adhesion proteins. Incorporating natural products or biotherapeutics may simultaneously reduce the side effects of contrast agents, achieve targeted therapy, and support imaging. For example, Zhang Mei et al. developed a nanomedicine for targeted diagnosis and treatment of AS plaques. Nanoparticles constructed from ***EGCG***, formaldehyde, and L-arginine retained the antioxidant properties of EGCG. Further modification with Mn^2+^ and a DKK1 antibody yielded favorable relaxivity, enabling plaque-specific MRI enhancement, whereas Cy5 labeling enabled plaque-specific *in vivo* imaging. This nanomedicine exhibited good biosafety, delayed plaque development, and improved plaque stability, supporting its potential for precise targeting and treatment of AS plaques (Zhao et al., 2018).

Studies by Zhang Shi et al. have shown that accurate detection of the progression of vulnerable AS plaques is important for risk stratification (Shi et al., 2024). In recent years, superparamagnetic iron oxide nanoparticles (IONs) have become a major focus in AS plaque detection as they generate characteristic hypointense signals on T2-weighted (T2W) MRI. To reduce potential adverse reactions to IONs in human subjects, a current clinical strategy is to integrate them into ligand-conjugated nanomedicines, such as nanoliposomes and biodegradable polymer nanoparticles. Despite significant progress, the sensitivity and signal-to-noise ratio (SNR) of IONs on T2W images under conventional magnetic field strengths remain limited, restricting their utility for high-precision cardiovascular imaging. To address this limitation, multimodal imaging approaches have emerged. These strategies combine two or more imaging modalities, such as MRI with nuclear medicine imaging, CT imaging, or fluorescence imaging, thereby improving imaging specificity and resolution. In this context, the research team designed a theranostic, lipid-coated NP that co-delivers a combination of imaging agents (specifically 125I-labeled IONs and curcumin) and achieves specific targeting of inflammatory macrophages within plaques via “eat-me” signals (phosphatidylserine, PtdSer, and 9-CCN) incorporated into the lipid membrane. Following macrophage internalization, curcumin is released into the cytoplasm, and the hybrid imaging agent is phagocytosed, enabling detection through hypointense signals on T2W images and the radioactive signal in single-photon emission computed tomography (SPECT). This multifunctional NP offers a promising platform for improving the current diagnosis and treatment of AS plaques and their complications.

### 5.3 Application and challenges of nanodelivery for TCM compound preparations

The TCM compound preparations, characterized by their “multi-component, multi-target, and multi-pathway” synergistic effects, have demonstrated unique advantages in the treatment of complex diseases such as AS. However, their clinical application has long been limited by issues including low solubility, poor stability, and insufficient bioavailability of active ingredients. The introduction of nanodelivery systems provides a new technical approach to overcome these limitations. By encapsulating TCM active ingredients in liposomes, polymer NPs, or biomimetic carriers, pharmacokinetic properties can be improved, lesion targeting can be enhanced, and systemic toxicity can be reduced (Fu et al., 2025). However, the complex chemical composition of TCM compounds presents challenges that extend beyond those of single-compound systems for the development of nanocarriers: First, the physicochemical properties of individual components differ substantially (e.g., a wide range of Log P values), and conventional carriers often fail to efficiently encapsulate both hydrophilic and hydrophobic components simultaneously. This can result in differential retention, where some components are well encapsulated while others are rapidly released. Addressing this requires the development of hybrid carriers, core–shell structures, or other advanced co-delivery systems. Second, maintaining the traditional “king, minister, assistant, and agent” ratio is central to TCM formulation design. Nanodelivery systems must therefore not only enable co-loading of multiple components but also preserve their relative proportions during *in vivo* circulation and release at the target site, placing stringent demands on carrier design and release kinetics. At the formulation evaluation level, the key quality attributes (CQAs) of TCM nanocarriers extend beyond conventional parameters such as particle size and zeta potential to include consistency of multi-component encapsulation, retention of component ratios, and interactions between the carrier and the components. Accurate determination of encapsulation efficiency (EE%) and drug loading (DL%) is further complicated by spectral overlap, challenges in separating free from encapsulated drugs, and ambiguity in defining “encapsulation.” These issues necessitate the establishment of dual quality control strategies combining fingerprint spectroscopy with multi-index analysis, alongside the use of high-sensitivity analytical techniques such as LC–MS/MS and separation methods including ultrafiltration and centrifugation (Zhang M. et al., 2025). Moreover, given the dynamic progression of AS, nanocarrier selection should align with disease stage. In early stages, endothelial protection and anti-inflammatory effects may be achieved through liposomes or biomimetic carriers for targeted delivery. In intermediate stages, polymer-based systems with sustained-release properties may support regulation of lipid metabolism, reflecting the gradual therapeutic modulation typical of TCM approaches. In advanced stages, where dense plaques are present, biomimetic strategies such as macrophage membrane-coated carriers may enhance penetration and targeting. From a safety perspective, long-term accumulation, potential toxicity, and immunogenicity of nanomaterials, particularly inorganic carriers, remain major barriers to clinical translation. It is essential to evaluate potential NP risks such as exacerbation of inflammation or induction of thrombosis, especially within the pathological context of endothelial dysfunction in AS patients. Future research should focus on building an “integrated treatment based on syndrome differentiation” intelligent nanodelivery system, integrating TCM syndrome classification with modern disease staging. This includes designing carriers aligned with TCM principles (e.g., “properties, flavors, and meridians”) and employing advanced manufacturing technologies such as microfluidics to improve batch consistency. Such efforts are expected to facilitate the transition of TCM nanocarriers from laboratory concepts to clinically viable products.

### 5.4 Pathological stage-matched nanocarrier selection for TCM formulas in AS: from early intervention to advanced plaque targeting

Since AS is a dynamic process involving endothelial injury, lipid deposition, inflammatory responses, and plaque formation and calcification, drug-delivery requirements vary significantly across pathological stages. Accordingly, selecting appropriate targeted nanocarriers is essential for maximizing the therapeutic performance of TCM compound prescriptions. To directly compare the strengths and limitations of different carriers, they can be evaluated in terms of drug-loading capacity, release kinetics, EPR-effect utilization, and *in vivo* safety (Zhang D. et al., 2025). Our in-depth analysis indicates that, whereas polymer microspheres and inorganic nanocarriers offer high drug loading, liposomes and biomimetic carriers better recapitulate the “multi-component, multi-target” features of TCM and enable sequential release. With respect to EPR-effect utilization, small-diameter liposomes and biomimetic carriers are more suitable for penetrating the endothelium at early lesions, whereas biomimetic carriers, owing to their intrinsic transendothelial migratory capacity, show greater potential for deep penetration into dense plaques at later stages. In terms of safety, liposomes and biomimetic carriers generally show the most favorable profiles since they originate from biological materials; polymer microspheres warrant attention to potential long-term effects of degradation products, and inorganic carriers, given ongoing controversies regarding biosafety, require additional caution (Li et al., 2024). Based on these comparisons, we propose the following suggestions for different types of TCM compound prescriptions: For prescriptions primarily targeting early-stage AS, aiming at endothelial protection and anti-inflammation (e.g., those containing danzhenone and chuanxiongine), liposomes or biomimetic carriers should be prioritized to achieve precise targeting while minimizing stimulation of normal blood vessels. For prescriptions targeting mid-stage AS, aiming at regulating lipid metabolism and inhibiting foam-cell formation (e.g., those containing hawthorn flavonoids), polymer microspheres are recommended because their high drug loading and sustained-release behavior can emulate the TCM therapeutic concept of “gradual dissipation and gradual dispersion.” For prescriptions targeting late-stage AS, requiring deep intraplaque penetration to inhibit calcification (e.g., those containing safflower and ligustrazine), biomimetic nanocarriers coated with macrophage or mesenchymal stem cell membranes merit particular exploration, given their strong homing and penetration capabilities that may enable more precise delivery. Overall, nanocarrier selection should be aligned with the pathological stage of AS and the core therapeutic actions of the TCM compound prescription. Future work should focus on developing an “individualized treatment” intelligent nanodelivery system by designing carriers that match the “properties and indications” of TCM compound prescriptions and contemporary medical staging, thereby enabling individualized and precise therapy.

### 5.5 Safety and nanotoxicology: from basic assessment to clinical translation challenges

Whereas nanocarriers have created unprecedented opportunities for treating AS with TCM, their biosafety, particularly potential toxicity with long-term use, remains the “last-mile” barrier to clinical translation. Most studies still emphasize efficacy, whereas toxicological characteristics of nanomaterials in the AS-specific pathological microenvironment, characterized by endothelial dysfunction, coagulation imbalance, and chronic inflammation, remain insufficiently investigated (Zhu et al., 2024). NPs may aggravate endothelial damage by generating catalytic ROS and disrupting eNOS function, increase thrombotic risk by activating the coagulation cascade and promoting platelet aggregation, and with long-term administration, non-degradable carriers may accumulate in the mononuclear phagocyte system and elicit immunogenicity-associated accelerated clearance. Of particular concern, TCM nanodelivery systems may concentrate trace toxic components at plaque sites or impair macrophage function via a “Trojan horse” effect, instead of achieving “detoxification and efficacy enhancement.” Therefore, future research should establish a multi-level assessment framework encompassing endothelial function, the coagulation system, and plaque stability; explicitly evaluate consequences of long-term low-dose exposure; prioritize the development of biodegradable biomimetic carriers; and integrate modern toxicological indicators with TCM toxicological theories to provide a robust safety basis for clinical translation of TCM nanodelivery systems (Feng et al., 2023).

## 6 Summary and outlook

AS is a chronic inflammatory disease associated with substantial mortality worldwide. This review summarizes recent advances in TCM nanomaterials for improving the diagnosis and treatment of AS and highlights the advantages and therapeutic potential of TCM nano-formulations for cardiovascular diseases, providing new strategies for prevention and management (Qian and Kai, 2020). By increasing drug solubility, improving bioavailability, and enabling targeted delivery, TCM nano-formulations may improve the efficacy of TCM in treating cardiovascular diseases and enhance patients’ quality of life. Nevertheless, current TCM nano-formulations remain limited by complex manufacturing, challenges in quality control, insufficient safety evaluation, and high costs (Stirrat et al., 2016).

Nanomaterials play a crucial role in improving therapeutic outcomes in AS. As summarized in the review, numerous TCM therapeutic agents have been incorporated into delivery systems to achieve anti-atherosclerotic efficacy (Ai et al., 2024). These agents include clinically used TCM drugs, anti-atherosclerotic peptides, anti-inflammatory cytokines, growth factors, and nucleic acids. Furthermore, the therapeutic mechanisms by which these agents treat AS include lowering cholesterol levels, enhancing cholesterol efflux, repolarizing macrophage phenotypes, promoting Treg differentiation, increasing pro-efferocytic activity, inducing autophagy in SMCs, modulating SMC phenotypes, inhibiting cell adhesion, proliferation, and migration, resolving local inflammation, and reducing matrix metalloproteinase (MMP) production (Qu et al., 2024). Future research should focus on combining nanomaterials with natural metabolites possessing anti-inflammatory or antioxidant properties (Yan et al., 2023).

Among the TCM nanomaterials discussed as delivery systems, curcumin NPs are the most extensively studied for treating AS. Curcumin is a natural polyphenol extracted from turmeric and has been identified as a potential modulator for shifting pro-inflammatory M1 macrophages toward the M2 phenotype. Its remarkable antioxidant, anti-inflammatory, anticoagulant, and lipid-lowering properties render it a promising candidate for targeting chronic inflammatory diseases. Other nanomaterials have also shown therapeutic potential, as summarized in Table 1.

**TABLE 1:** Nanodrug formulations of TCM metabolites and their R&D advantages.

No.	TCM Com	Source	2D-Structure	Nano-dosage form	Advantages	References
1	Curcumin (Cur)	(from Curcuma longa L.)	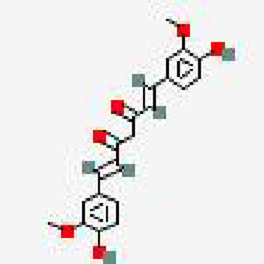	Curcumin nanoparticles encapsulated by linear-dendritic monomethoxy polyethylene glycol-polycaprolactone; biomimetic nanocarriers; novel synthesis of quercetin nanoparticles (N-QCT) encapsulated by solid lipid nanoparticles (SLNs); HA-M@P@(Evol+Cur) nanoparticles; enhanced solubility	Improved bioavailability and targeting, while retaining anti-inflammatory properties	Shamsi et al. (2024), Shahbazi et al. (2018), Fontana et al. (2024), Liu et al. (2025b), Shi et al. (2024)
2	Tanshinone IIA	Salvia miltiorrhiza Bunge	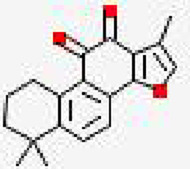	Peptide-based recombinant high-density lipoprotein pHDL nanocarriers with plaque-targeting effect	PHDL not only exerts a therapeutic effect on AS by itself, but also serves as a carrier to provide storage space for fat-soluble drugs, featuring high-efficiency drug-carrying capacity, which can enhance the tissue targeting of drugs and reduce the interference of drugs in non-lesion areas	Xiao et al. (2025), Gill et al. (2021)
3	Colchicine	Colchicine (from Colchicum autumnale L.)	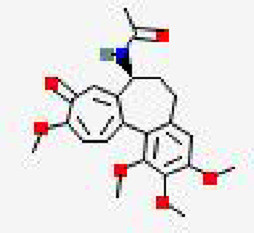	Methylated β-cyclodextrin polymer nanoparticles	Targeted inhibition of the NLRP3 inflammasome, blocking the release of pro-inflammatory factors, inhibiting the inflammatory storm in the vascular wall, and improving its bioavailability and targeting ability	Shamsi et al. (2024), Li et al. (2024)
4	Notoginsenoside R1	Notoginsenoside R1 (from Panax notoginseng (Burkill) F.H.Chen)	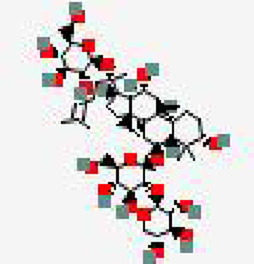	CD11b antibody was conjugated to mesoporous silica nanoparticles (MSN) loaded with notoginsenoside R1 to construct MSN-NGR1-CD11b antibody nanoparticles	Enhance the myocardial targeting efficiency of notoginsenoside R1 nanoparticles, with the drug concentration in the target tissue being higher than that of ordinary formulations	Yin et al. (2020)
5	Epigallocatechin Gallate (EGCG)	Epigallocatechin Gallate (from Camellia sinensis (L.) Kuntze)	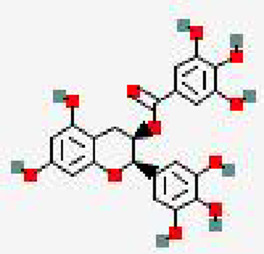	Polymer nanoparticles constructed with epigallocatechin gallate (EGCG), formaldehyde, and L-arginine	Retains the antioxidant properties of epigallocatechin gallate (EGCG)	Shao et al. (2025), Zhao et al. (2018)
6	Paclitaxel (PTX)	Paclitaxel (from Taxus brevifolia Nutt.)	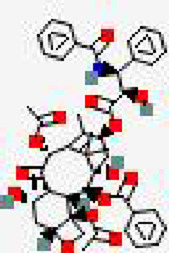	Delivery of vascular endothelial growth factor (VEGF) plasmids via PLGA nanoparticles (NPs) combined with paclitaxel (PTX)	Improved bioavailability and targeting	Xu et al. (2023)
7	Ferulic Acid (FA)	Ferulic Acid (FA) Ferulic Acid (from Ferula assa-foetida L.)	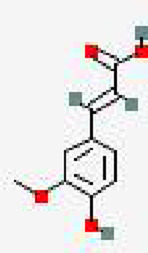	Composed of a ferulic acid-based polyester core and a shell modified with amphiphilic molecules for targeting scavenger receptors	Improved bioavailability and targeting	Kim et al. (2023)
8	Gingerol	Gingerol (Wang et al., 2020)-Gingerol (from Zingiber officinale Roscoe)	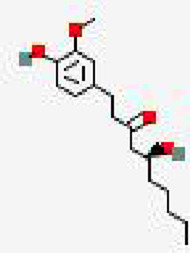	Garlic-derived exosome-like nanovesicles	Reduces the expression of CD36 and LOX-1 scavenger receptors in macrophages, and inhibits the uptake of ox-LDL by macrophages	Zhu et al. (2023)
9	sulforaphane	Sulforaphane (from *Brassica oleracea* L.)	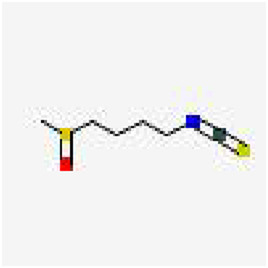	Carbon dots obtained from broccoli water extract (BWE)	Improved bioavailability and targeting	Deng et al. (2023)
10	Quercetin	Quercetin (from many plants, e.g., Allium cepa L.)	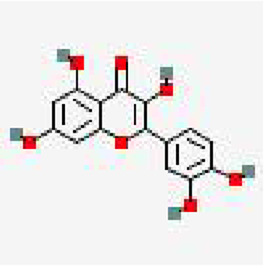	Inhalable quercetin-alginate nanogel (QCT-alginate nanogel, QUNG))	Significantly improved the solubility and oral bioavailability of QCT	Chen R. et al. (2022), Shamsi et al. (2024)
11	Cineole	Cineole Cineole (from Eucalyptus globulus Labill.) Eucalyptus globulus: https://powo.science.kew.org/taxon/urn:lsid:ipni.org:names:592874-1	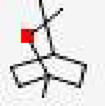	Oral nanoemulsions stabilized by polysaccharide-protein complexes	Enhanced the *ex vivo* and *in vivo* stability of cinobufagin (CIN), prolonged its retention time in the gastrointestinal tract, strengthened the ability of CIN to penetrate the mucus layer and intestinal epithelial cells, and simultaneously increased the oral bioavailability and plaque deposition of CIN	Chen et al. (2023b)
12	Ginsenoside	Ginsenoside Ginsenoside (from Panax ginseng C.A.Mey.) Panax ginseng: https://powo.science.kew.org/taxon/urn:lsid:ipni.org:names:77211633-1	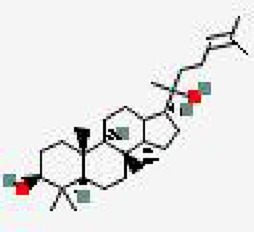	Carrier-free nanoparticles; saponin-encapsulated ultrasound microbubble system (Rb3NPs@MBs)	Exhibits excellent abilities in eliminating intracellular ROS, inhibiting the secretion of pro-inflammatory factors, and suppressing intracellular lipid deposition *in vitro*; the microbubble system effectively encapsulates saponin Rb3 nanoparticles and promotes their targeted accumulation in the aortic arch of AS mice through ultrasound-targeted microbubble destruction (UTMD)	Yan (2024), Zhong et al. (2025)
13	Shikonin	Shikonin Shikonin (from Lithospermum erythrorhizon Siebold and Zucc.)	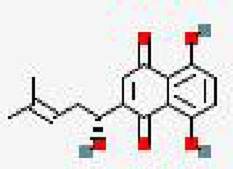	Shikonin nanogel; dual-targeted biological liposomal nanomedicine loaded with shikonin (SKN) and evolocumab (Evol) (HA-ML@ES NPs)	With high drug-loading efficiency; prolongs the half-life of drugs and effectively co-delivers drugs to damaged endothelial cells and inflammatory macrophages at AS plaque sites	Li X. et al., (2023), Zhang et al. (2025b)
14	Andrographolide	Andrographolide Andrographolide (from Andrographis paniculata (Burm.f.) Nees)	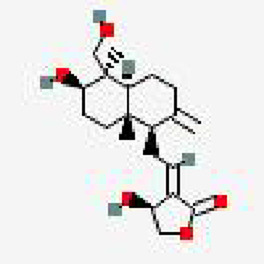	PEG-block-polypropylene sulfide (PEG-b-PPS) micelles	These micelles respond to the oxidative microenvironment and can deliver andrographolide to improve its low solubility	Wu et al. (2018)
15	Celastrol	Celastrol (from Tripterygium wilfordii Hook.f.)	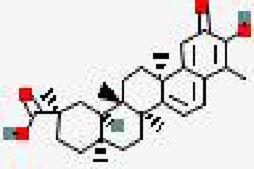	ROS-responsive PEG-b-PSS micelles are used to deliver celastrol	PEG-b-PSS micelles can significantly improve the therapeutic effect of hydrophobic therapeutic agents and exhibit high loading efficiency	Allen et al. (2019)
16	Berberine (BBR)	Berberine (BBR) Berberine (from Berberis vulgaris L.)	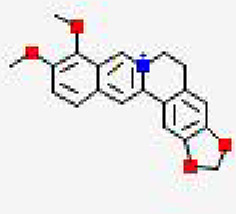	Micelles based on d-α-tocopheryl polyethylene glycol succinate loaded with the plant drug berberine (BBR); proliposomes (PL) loaded with BBR	Improve the oral bioavailability and therapeutic effect of BBR; enhance its bioavailability and targeting ability	Man et al. (2022), Duong et al. (2022), Ma et al. (2020), Wu et al. (2024)
17	Artemisinin (ART)	Artemisinin (ART) Artemisinin (from Artemisia annua L.) Artemisia annua: https://powo.science.kew.org/taxon/urn:lsid:ipni.org:names:304682-2	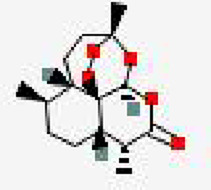	M@PB@(PC+ART) NPs; nanocomposites co-loaded with artemisinin (ART) and procyanidin (PC)	Increase the recruitment of NPs to the plaque area and prolong the circulation time	Zhou et al. (2022)
18	Procyanidin (PC)	Procyanidin (PC) Procyanidin (from Vitis vinifera L.)	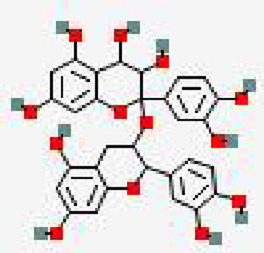	M@PB@(PC+ART) NPs, nanocomposites co-loaded with artemisinin (ART) and procyanidin (PC); hyaluronic acid (HA)-modified HA@PC@Pita NPs that combine the advantages of pitavastatin (Pita) in efferocytosis recovery and procyanidin (PC) in cholesterol efflux enhancement	Increase the recruitment of NPs to the plaque area and prolong the circulation time; HA@PC@Pita NPs can induce M1/M2 repolarization and upregulate the expression of ERK5/Mertk to restore the efferocytotic function of macrophages	Zhou et al. (2022), Wu Y. et al., (2025)
19	Resveratrol (RV)	Resveratrol (RV) Resveratrol (from Vitis vinifera L.)	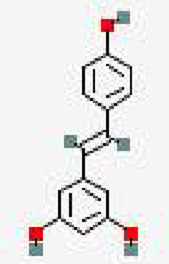	HMLRPP NPs: Pitavastatin-loaded poly (lactic-co-glycolic acid) (PLGA) nanoparticles (NPs) and resveratrol-loaded liposomes (Lipo) serve as the core, coated with macrophage membranes and modified hyaluronic acid (HA)	HMLRPP NPs can continuously release pitavastatin (Pit) and resveratrol (Res) in an acidic environment, and alleviate hyperhomocysteinemia (HHcy)-induced AS (AS) by inhibiting macrophage ferroptosis, reducing inflammation, and ameliorating lipid dysregulation	Yang et al. (2025)
20	Mulberry	Mulberry Mulberry (Morus alba L.)	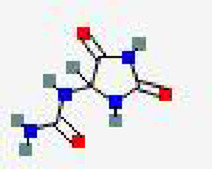	Extracellular vesicle-like nanoparticles (MFEVLPs)	Improved bioavailability and targeting	Sui et al. (2025)
21	HSYA	Hydroxysafflor Yellow A (from Carthamus tinctorius L.)	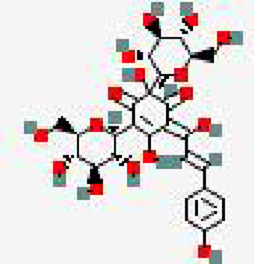	HA-ML@(H+R)NPs	The biomimetic system effectively delivers hydroxysafflor yellow A (HSYA) + resveratrol (RV) to plaque sites, significantly enhancing plaque stability, reducing blood lipids, and lowering the levels of inflammatory cytokines	Dong et al. (2025)
22	Ginger	Ginger Ginger (Zingiber officinale Roscoe) Zingiber officinale: https://powo.science.kew.org/taxon/urn:lsid:ipni.org:names:798372-1 −7	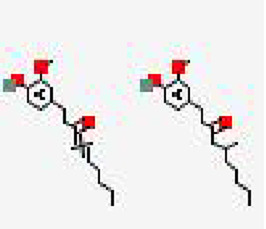	Exosome-like nanovesicles	Inhibit low-density lipoprotein (LDL) oxidation and reduce foam cell formation	Zhu and He (2023)
23	Puerarin (PU)	Puerarin (PU) Puerarin (from Pueraria montana var. lobata (Willd.) Sanjappa and Pradeep)	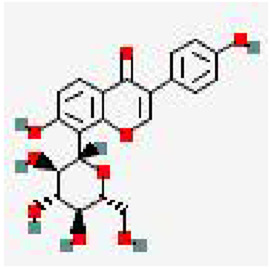	RGCP@polyurethane nanoparticles	Significantly reduce oxidative stress and inflammation levels, and exhibit excellent biocompatibility *in vivo*	Liu et al. (2025a)

Among the nanomaterials investigated, HDL NPs and their encapsulated therapeutic molecules can produce synergistic effects (Tang J. et al., 2021). Beyond HDL NPs, established platforms, including polymeric NPs, dendrimers, micelles, and liposomes, have also been investigated to enhance the efficacy of their payloads. LNPs, considered among the TCM delivery systems with the greatest potential for clinical translation, and PEG-based micelles have been widely studied for treating AS owing to their simple structures and readily modifiable lipid tails (Wang et al., 2022). Research on polymeric NPs has focused primarily on HA and PLGA NPs; therefore, future work should expand to carbon-based nanoparticles (CDNPs), extracellular vesicles, biomimetic NPs, and inorganic NPs. Improving drug loading into extracellular vesicles and developing novel extracellular vesicles derived from stem cells, as well as biomimetic NPs for AS treatment, may become key directions in the future development of TCM nano-formulations (Zhang M. et al., 2022). Furthermore, drug-loaded nanosystems coated with CMs, which can enable sustained delivery and targeted lesion homing, are also an active area of research. In addition, hydrophilic materials such as PEG and cyclodextrin (CD) can impart stealth properties to NPs, helping them evade opsonin adhesion, where opsonins are key proteins that enhance phagocytic cell function.

However, biological barriers remain a key determinant of nanomedicine delivery in AS. After entering the bloodstream, NPs encounter multiple barriers that can reduce plaque-specific bioavailability and limit the practical performance of antiplatelet therapeutic and diagnostic approaches (Liu Y. et al., 2022). Accordingly, nanoparticle design must address two major challenges: minimizing non-specific uptake and clearance resulting from the mononuclear phagocyte system (MPS) and overcoming physiological barriers associated with AS progression. For anti-atherosclerotic NPs to reach plaques, rapid clearance is a primary obstacle (Wu B. et al., 2025; Tu et al., 2022; de La’zaro and Mooney, 2021). NPs with diameters outside the ∼5–150 nm range, or with a positive surface charge, are more susceptible to renal filtration or non-selective sequestration by hepatic macrophages (MPS) (Zhou et al., 2020). Precise regulation of particle size, shape, and surface charge is therefore essential to reduce premature clearance. In addition, oral biomimetic targeted delivery systems and “Trojan horse” NPs may help mitigate toxicity risks resulting from non-specific distribution.

The treatment model for AS has evolved from simply controlling risk factors to targeted plaque intervention. However, current plaque-targeting strategies that use recombinant high-density lipoprotein (rHDL) as a carrier remain difficult to promote due to system complexity, limited controllability, and high cost (Chen H. et al., 2022). Although the aforementioned nanoplatforms demonstrated therapeutic potential in preclinical studies, it must be pointed out that there are several key limitations in the methodology and evidence strength of the existing literature, which require caution when interpreting the results. Specifically: For literature (Tang J. et al., 2021; Wang et al., 2022; Zhang M. et al., 2022) (HDL nanoparticles’ synergistic effect): Although this study proposed the hypothesis of synergy, its sample size was small and no efficacy analysis was conducted, thus the stability and generalizability of the effect size are questionable. Additionally, the study did not use blind methods to evaluate the outcome indicators, which may introduce detection bias. For literature (Liu Y. et al., 2022): The causal inference in this article is relatively weak. The authors directly attributed the *in vitro* macrophage polarization effect to the anti-inflammatory efficacy *in vivo*, but did not provide clear evidence of the pharmacokinetic-pharmacodynamic (PK-PD) link, nor did they rule out the non-specific immune modulation caused by the nanocarriers themselves. The summary statements about the nature of the review literature lack direct experimental support and belong to forward-looking speculation rather than data-based conclusions. The original studies cited in the text mostly used highly heterogeneous preparation methods (such as insufficient purity from ultracentrifugation), making comparisons between different studies lack a benchmark, and future standardized characterization protocols are needed. In conclusion, the current evidence base for the use of traditional Chinese medicine nanomaterials in the treatment of atherosclerosis is still at an early stage. Most studies have significant room for improvement in terms of methodological rigor (randomization, blinding, sample size calculation) and the conservatism of their conclusions. Meanwhile, whereas many TCM active components exhibit excellent effects in preventing and treating AS, their poor water solubility limits clinical application. Recent advances in TCM nano-formulations have achieved breakthroughs in cardiovascular targeting through precise surface modification. Biomimetic peptide-functionalized HDL (pHDL) nanocarriers use peptides to replace full-length apolipoprotein A-1 (apoA-1), and microfluidic assembly enables tight process control to enhance plaque-targeting efficiency, thereby increasing the local concentration of tanshinone within plaques (Zhang et al., 2020). For example, Ning et al. developed a TCM nano–targeted delivery system for atherosclerotic plaques by using microfluidics to precisely regulate fluid behavior at the micro- and nano-scales and by replacing full-length proteins with lower-cost peptides. They prepared peptide-based pHDL nanocarriers capable of active plaque targeting and recruitment. Ning and Ruodan’s team at the China Academy of Chinese Medical Sciences further used microfluidic chip technology to prepare pHDL NPs loaded with multiple TCM components, including tanshinone IIA and berberine. Nano-formulations produced using this approach showed high yield, uniform particle size, good reproducibility, and effective plaque targeting (Gao et al., 2022).

Future research should prioritize these directions and emphasize effective clinical translation. A key avenue is the development of theranostics that leverage the therapeutic effects of natural products or biotherapeutics (Bangari et al., 2023). It is also important to design nanomaterials for the co-delivery of two therapeutic agents to plaques and to evaluate potential synergy between the paired agents. Furthermore, as discussed earlier in this review, most reported theranostic nanomaterials are designed to target macrophages or endothelial cells; however, plaques also contain large numbers of T cells and B cells, as well as calcifications (Smit et al., 2023). Accordingly, the potential of natural nanomaterials modified with ligands that target these additional plaque components should be explored to improve both imaging performance and therapeutic efficacy in AS. Before the clinical translation of the various therapeutic strategies reviewed above, the safety and efficacy of nanomaterials intended for human use must be rigorously evaluated, as clinical development must balance reproducible efficacy with patient safety (Yang et al., 2022). In addition, establishing good manufacturing practice (GMP) for nanomaterial production and strengthening interactions between small laboratories and the pharmaceutical industry are essential (Jiang et al., 2020). Future research should also emphasize large-scale manufacturing, improved stability, and long-term storage of nanomaterial-based therapeutics, contrast agents, and theranostics to reduce the risk of failure during clinical translation.

To assess the quality of the included primary literature, we evaluated the reporting of critical information against the ConPhyMP guidelines (Supplementary Table S1) (Heinrich et al., 2022). Our analysis revealed significant reporting gaps: although nearly all studies correctly reported the plant species, key information, such as voucher specimen deposition and extraction details (e.g., drug-to-solvent ratio, temperature, and duration), was frequently omitted. This lack of transparency substantially impairs reproducibility and comparability in TCM nanoformulation research and underscores the need to adopt standardized reporting practices. A critical evaluation of the analytical methods employed in the included studies against the ConPhyMP guidelines also revealed major deficiencies (Supplementary Table S2). Although most studies investigated pharmacopoeial-grade botanical drugs (Type A extracts), compliance with pharmacopoeial standards was rarely verified. Moreover, most studies quantified only a single marker compound using HPLC-UV, with little evidence of multi-component fingerprinting. This reductionist approach does not capture the chemical complexity of TCM formulas and severely limits the reliability of subsequent encapsulation-efficiency measurements and the interpretation of pharmacological data. These observations further highlight the need for more comprehensive analytical workflows to ensure that nano-formulation research is built upon a well-defined and reproducible chemical foundation.

Research and development of TCM nano-formulations require further strengthening through enhanced interdisciplinary collaboration, with experts in materials science, pharmacy, and medicine working together to optimize preparation processes, improve reproducibility and stability of TCM nano-formulations, and reduce production costs. Establishing a robust quality-control framework, including unified quality standards and detection methods, is essential to ensure reliable product quality (Zhang et al., 2021). In-depth safety studies are also needed to clarify *in vivo* metabolism and toxicity mechanisms of nano-formulations, thereby supporting clinical application (Liu H. et al., 2022). With continued scientific advances and technological maturation, TCM nano-formulations are expected to achieve further breakthroughs in cardiovascular therapy and may become an important modality for treating cardiovascular diseases.
