# Supplementary material for: Emerging nanotechnological strategies for delivering bioactive components from botanical drugs and traditional Chinese medicine in atherosclerosis therapy: from formulation to mechanism
Source: Front Pharmacol. 2026 May 26;17:1792534. doi: 10.3389/fphar.2026.1792534 (PMC13246730; doi:10.3389/fphar.2026.1792534)
Supplement: Supplementary file 1 [file DataSheet2.pdf]

Supplementary Table 2: Assessment of analytical methods in included studies based on ConPhyMP Table 4

NO.	Assessment Item	Findings from assessed studies	Relevant discussion in our review
1	Extract Type	The extracts discussed in this review were mainly derived from pharmacopoeia- recorded herbs such as Salvia miltiorrhiza, Curcuma longa, and Scutellaria baicalensis, and can therefore be classified as Type A extracts.	See Section 6 "Quality and Limitation Analysis of Included Studies"
2	Primary/Principal Method (Pharmacopoeia Compliance)	Main issue: Although pharmacopoeial standards exist for these herbs, most original studies only briefly mentioned the use of a certain extract without explicitly stating whether its preparation complied with pharmacopoeial requirements or providing the assay results of pharmacopoeia- specified marker compounds. This made it impossible to verify whether the extract quality was consistent and reproducible.	See Section 6 "Quality and Limitation Analysis of Included Studies"
3	Primary/Principal Method (Fingerprint and Multi- component Quantitation)	Main issue: It was found that the vast majority of studies only used HPLC- UV for quantitation of a single marker compound (e.g., tanshinone IIA, curcumin). Very few studies employed three orthogonal fingerprint methods for holistic characterization of complex extracts. For compound preparations, studies determining multiple marker components simultaneously were extremely rare. This led to a very one- sided understanding of the extract chemical composition and failed to ensure that the "multi- component" property was preserved and accurately evaluated in nanoformulations.	See Section 6 "Quality and Limitation Analysis of Included Studies" and Section 5 "Challenges in EE% and DL% Determination"
4	Alternative Methods	Considering the practical conditions in most laboratories, using a single method (e.g., HPLC- UV) combined with multiple detection parameters is a feasible alternative. However, few studies reported detection at different wavelengths (e.g., 210 nm, 254 nm, 360 nm) to cover more diverse compound classes .	See Section 6 "Quality and Limitation Analysis of Included Studies"
5	Use of Reference	Most studies using single compounds employed	-

NO.	Assessment Item	Findings from assessed studies	Relevant discussion in our review
	Standards	commercial reference standards, ensuring quantitative accuracy. However, for compound preparations, there was a lack of comparison with fingerprint profiles of standard extracts.	
6	Comparison of Different Samples	This item mainly applies to method development and was rarely addressed in the original studies.	-
