# Supplementary material for: Emerging nanotechnological strategies for delivering bioactive components from botanical drugs and traditional Chinese medicine in atherosclerosis therapy: from formulation to mechanism
Source: Front Pharmacol. 2026 May 26;17:1792534. doi: 10.3389/fphar.2026.1792534 (PMC13246730; doi:10.3389/fphar.2026.1792534)
Supplement: Supplementary file 2 [file DataSheet1.pdf]

Supplementary Table 1: Assessment of included studies based on ConPhyMP Table 3

No.	Assessment Item	Findings from assessed studies)	Relevant discussion in our review
1	Title and Abstract	Most included studies had acceptable titles and abstracts, which clearly indicated the plant materials and therapeutic directions.	-
2	Herbal Medicine and Taxonomic Authentication of the studies to a certain extent.	Main issue: Although all studies provided the Latin names of plant species (e.g., Salvia miltiorrhiza), the vast majority did not report the deposition information of voucher specimens, nor did they specify who performed the identification and by which method. This compromises the reproducibility	See Section 6 "Quality and Limitation Analysis of Included Studies"
3	Description of Extraction and Extraction	Main issue: The reporting quality was inconsistent across studies. Most studies reported the extraction solvent, but key parameters such as drug-solvent ratio, extraction temperature and duration were often missing. For compound preparations, very few studies reported the exact proportions of each herb, posing a major challenge to the standardization of compound formulations.	See Section 6 "Quality and Limitation Analysis of Included Studies" and Section 5 "Challenges in the Co-delivery of Traditional Chinese Medicine Compounds"
4	Legal Basis for Collection	None of the included studies mentioned compliance with the Nagoya Protocol or phytosanitary regulations. This is acceptable given the experimental nature of these studies.	-
5	Description of Finished (Commercial) Products	Most studies used laboratory- made extracts, so this item was not applicable. A small number of studies using commercial products generally provided the product name and batch number.	-
